# Discovery and evaluation of active compounds from Xuanfei Baidu formula against COVID-19 via SARS-CoV-2 M^pro^

**DOI:** 10.1186/s13020-023-00790-0

**Published:** 2023-08-02

**Authors:** Min Zhang, Liting Liu, Yao Zhao, Yipeng Cao, Yan Zhu, Lifeng Han, Qi Yang, Yu Wang, Changjian Wang, Han Zhang, Yuefei Wang, Junhua Zhang

**Affiliations:** 1 State Key Laboratory of Component-Based Chinese Medicine, Tianjin University of Traditional Chinese Medicine, 10 Poyanghu Road, West Area, Tuanbo New Town, Jinghai District, Tianjin, 301617 China; 2 Evidence-Based Medicine Center, Tianjin University of Traditional Chinese Medicine, Tianjin, 301617 China; 3 Tianjin Key Laboratory of TCM Chemistry and Analysis, Tianjin University of Traditional Chinese Medicine, Tianjin, 301617 China; 4 Key Laboratory of Pharmacology of Traditional Chinese Medical Formulae (Ministry of Education), Tianjin University of Traditional Chinese Medicine, Tianjin, 301617 China; 5 Haihe Laboratory of Modern Chinese Medicine, Tianjin, 301617 China; 6 Shanghai Institute for Advanced Immunochemical Studies and School of Life Science and Technology, ShanghaiTech University, Shanghai, 200031 China; 7 National Supercomputer Center in Tianjin, Tianjin, 300457 China; 8 Guangzhou Laboratory, Guangzhou, 510005 China

**Keywords:** SARS-CoV-2, M^pro^, Xuanfei Baidu formula (XFBD), Acteoside

## Abstract

**Background:**

The coronavirus disease 2019 (COVID-19) caused by the severe acute respiratory syndrome coronavirus (SARS-CoV-2) is still a widespread concern. As one of the effective traditional Chinese medicine (TCM) formulae, Xuanfei Baidu formula (XFBD) shows significant efficacy for treatment of COVID-19 patients. However, its antiviral active compounds and mechanism are still unclear.

**Purpose:**

In this study, we explored the bioactive compounds of XFBD and its antiviral mechanism by integrating computational analysis and experimental testing.

**Methods:**

Focusing on the SARS-CoV-2 main protease (M^pro^), as a key target in virus transcription and replication, the fluorescence resonance energy transfer (FRET) assay was built to screen out satisfactory natural inhibitors in XFBD. The surface plasmon resonance (SPR) and isothermal titration calorimetry (ITC) were undertaken to verify the binding affinity of ligand-M^pro^. Omicron BA.1.1 and BA.2.3 variants were used to evaluate the antiviral activity of the focused compounds in non-cytotoxicity concentrations. For introducing the molecular mechanism, computational modeling and NMR spectra were employed to characterize the ligand-binding modes and identify the ligand-binding site on M^pro^.

**Results:**

From a library of 83 natural compounds, acteoside, licochalcone B, licochalcone D, linoleic acid, and physcion showed the satisfactory inhibition effects on M^pro^ with IC_50_ ranging from 1.93 to 42.96 µM, which were further verified by SPR. Showing the excellent binding affinity, acteoside was witnessed to gain valuable insights into the thermodynamic signatures by ITC and presented antiviral activity on Omicron BA.1.1 and BA.2.3 variants in vitro. The results revealed that acteoside inhibited M^pro^ via forming the hydrogen bond between *7-H* of acteoside and M^pro^.

**Conclusion:**

Acteoside is regarded as a representative active natural compound in XFBD to inhibit replication of SARS-CoV-2, which provides the antiviral evidence and some insights into the identification of SARS-CoV-2 M^pro^ natural inhibitors.

**Graphical Abstract:**

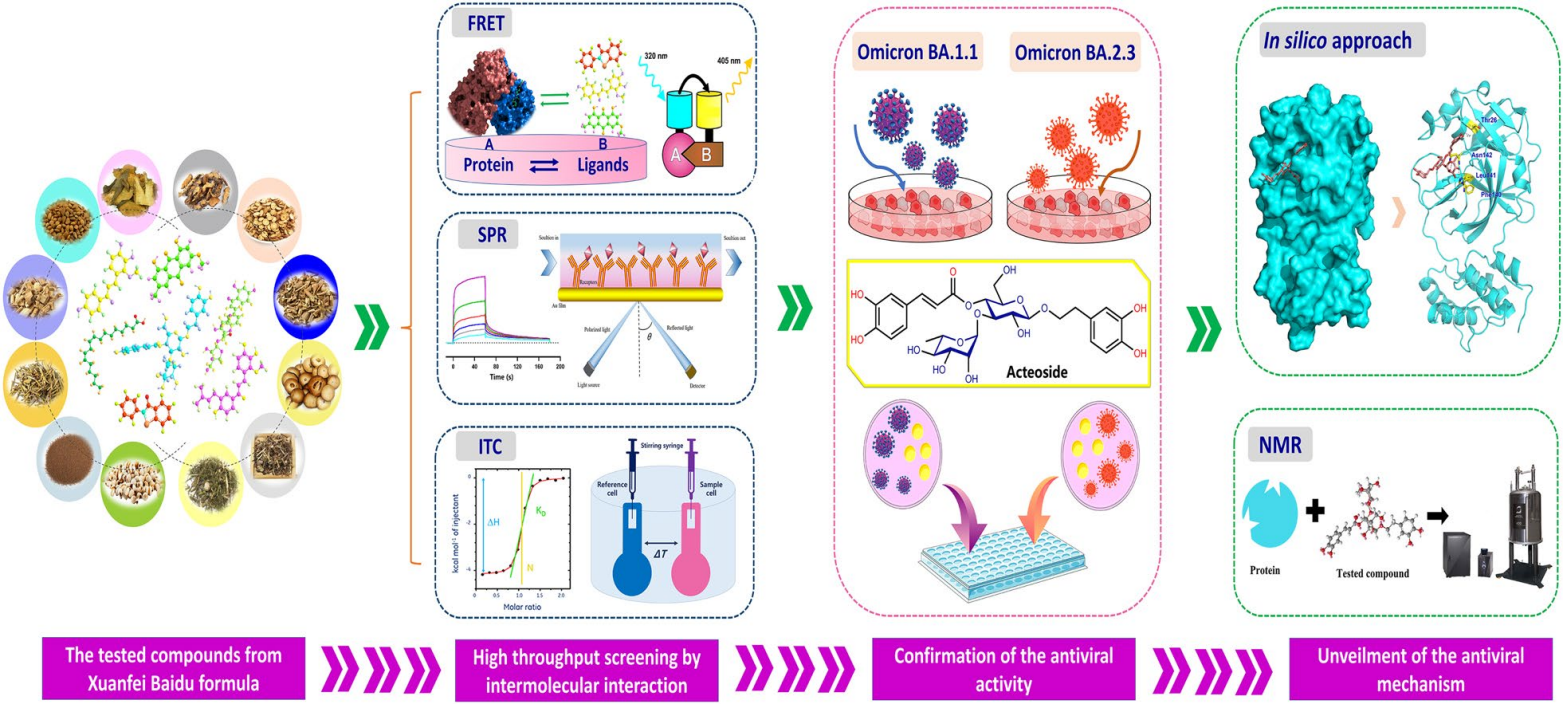

**Supplementary Information:**

The online version contains supplementary material available at 10.1186/s13020-023-00790-0.

## Introduction

The pandemic COVID-19 caused by SARS-CoV-2 has threatened public health all over the world for more than 3 years. As of May 10, 2023, more than 765 million cases have been confirmed, and over 6.92 million lethal cases have been reported by the World Health Organization (WHO) [1]. Due to the widespread practice of traditional Chinese medicine (TCM), the war against COVID-19 was equipped with the confident weapon to protect people’s health [2]. Numerous clinical evidences showed that TCMs relieve symptoms of COVID-19, block the worse condition, and reduce mortality [3, 4]. Xuanfei Baidu formula (XFBD), as one of the effective formulae in “three medicines and three prescriptions”, is recommended for treating COVID-19 patients in the 3rd–9th editions of the diagnosis and treatment protocols released by the National Health Commission of the People’s Republic of China. In March 2021, XFBD granules has been approved by National Medical Products Administration (NMPA) for treating COVID-19 patients in mild or moderate cases, which can relieve fever, cough, fatigue and other clinical symptoms, prevent the mild to the severe progression, and decrease mortality in clinical practice [5, 6]. Composed of 13 herbs (Additional file 1: Table S1), XFBD is modified from classical TCM formulae, such as Maxing Shigan decoction (MXSG), Maxing Yigan decoction (MXYG), Qianjin Weijing decoction (QJWJ), and Tingli Dazao Xiefei decoction (TLDZXF) [7], with indications of dispelling lung dampness, clearing heat and penetration evil, and purifying lungs and detoxification.

In previous studies, the network pharmacology analysis showed that the efficacy of XFBD are possibly originated from antiviral infection and recovery of lung injury [7, 8]. The pharmacological studies have verified that XFBD can suppress inflammatory response, and restore the balance of energy metabolism, which also can be effective in inhibition of macrophage infiltration and pulmonary fibrosis via IL-6/STAT3 signaling pathway [9], and reduction of acute lung injury by regulating infiltration of neutrophils and macrophages via PD-1/IL-17A pathway [10]. However, as an effective formula, the bioactive compounds in XFBD against SARS-CoV-2 were yet unveiled.

SARS-CoV-2 is a novel positive-sense single-stranded RNA virus belonging to the subfamily *β*-coronavirus. The virus genomes mainly encode four structural proteins and sixteen nonstructural proteins that control the process of virus invasion, replication, assembly, and release. As the key protease to orchestrate the replication and transcription of SARS-CoV-2, the main protease (M^pro^) is about 33.8 kDa, which consists of a polypeptide chain of 306 amino acids [11]. Given its low homology with mammalian proteins, M^pro^ is a satisfactory target for the development of chemical inhibitors with broad-spectrum antiviral activity [12]. Up to now, Paxlovid, S-217622, and DC402234 were reported as SARS-CoV-2 M^pro^ inhibitors in clinical trial phases or market [13], showing the dawn for the era of new inhibitors to be discovered.

**Fig. 1:**
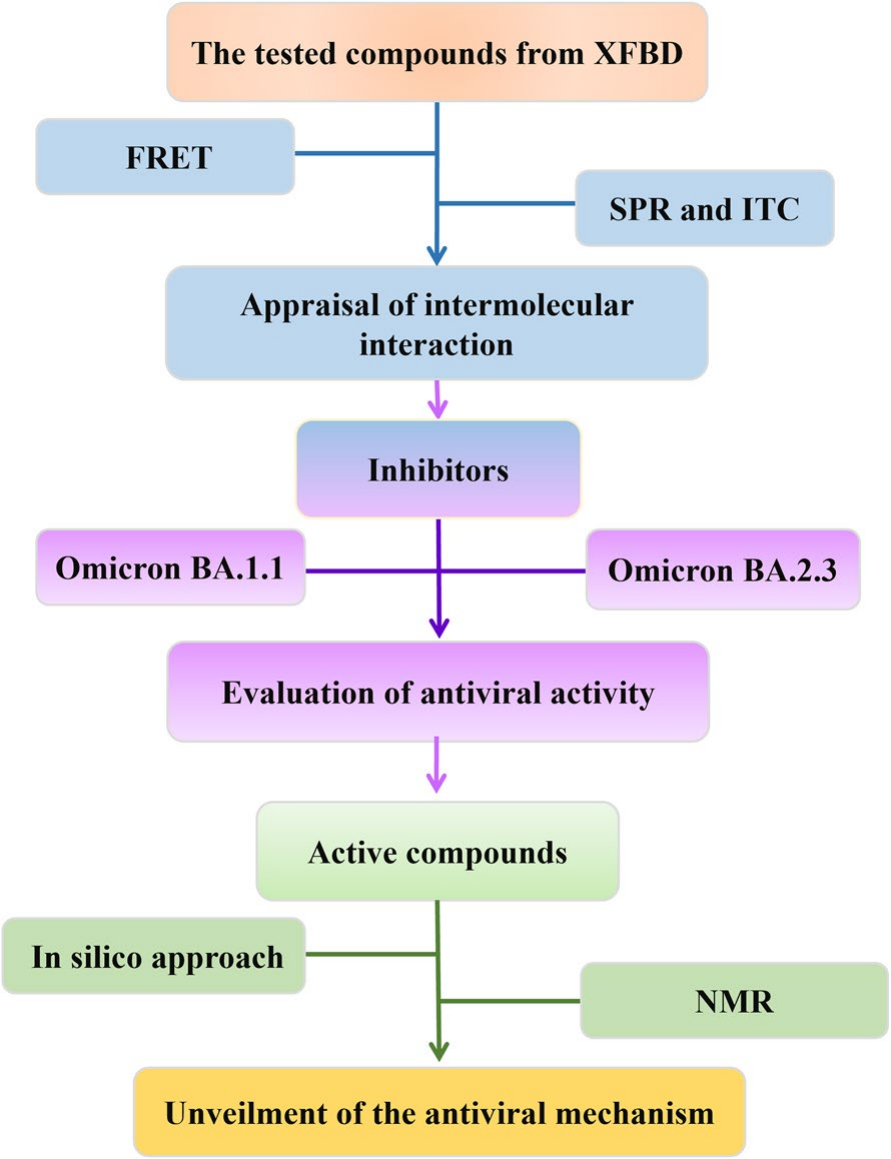
The flow chart for discovery and evaluation of active compounds against SARS-CoV-2 M^pro^

In this study, we focus on M^pro^ to explore the bioactive compounds and related antiviral mechanisms from the compounds library built from XFBD. To test the intermolecular interaction, the integrated methods have proved that acteoside, licochalcone B, licochalcone D, linoleic acid, and physcion were identified as the potential active compounds by fluorescence resonance energy transfer (FRET) and surface plasmon resonance (SPR). And the interaction of M^pro^-acteoside was further confirmed by isothermal titration calorimetry (ITC) to determine the thermodynamic parameters. Omicron BA.1.1 and BA.2.3 variants were employed to demonstrate the antiviral activity of acteoside in vitro. Molecular docking and molecular dynamics (MD) simulation suggested that the hydrogen bond between acteoside and M^pro^ was considered as the key interaction, which was demonstrated by nuclear magnetic resonance (NMR). Our study elucidated and evaluated the active compounds in XFBD against COVID-19 via inhibiting SARS-Cov-2 M^pro^ activity, which provides insights for discovering the active compounds from the tested formula and unveiling corresponding antiviral mechanism (Fig. 1).

## Material and methods

### Reagents and chemicals

Based on the preliminary screening results by network pharmacology analysis (Additional file 1: Fig. S1, S2, S3, S4, S5), the compounds library was built, containing 83 compounds, which were obtained from Shanghai Yuanye Biotechnology Co., Ltd. (Shanghai, China) with HPLC purity > 98% (Additional file 1: Table S2). PBS, Tris, DMSO, and imidazole were acquired from Solarbio (Beijing, China) and other commercial suppliers. The sensor Chip (CM5), acetate (pH 4.0), PBS-P, and the amine coupling kit were purchased from GE Healthcare Life Sciences (Boston, USA).

### ***Cloning, protein expression and purification of SARS-CoV-2 M***^***pro***^

The plasmid, which encodes the full-length gene of SARS-CoV-2 M^pro^ (NC_045512), was transferred into *Escherichia coli* BL21 (DE3), cultured in Luria broth medium with 100 µg/mL ampicillin at 37 ℃ for 6–8 h. Then, 500 µM IPTG was added into the cell culture flasks to induce the expression of M^pro^ for 16–20 h at 16 ℃ [14]. The cells were enriched and crushed through the high-pressure homogenizer, whose supernatant was collected and purified through Ni–NTA affinity column. The buffer at pH 8.0 (20 mM Tris–HCl, 150 mM NaCl, and 5% Glycerol) containing 20 or 300 mM imidazole was used to remove debris and elute M^pro^, respectively. The C-terminal 6 × His tag of M^pro^ was removed by human rhinovirus 3C protease, which was further subjected to purification by size exclusion chromatography (Superdex 200 Increase 10/300 GL). The purified M^pro^ was checked by SDS-PAGE with purity > 95%. At last, M^pro^ was preserved at – 80 ℃ in buffer (10 mM Tris–HCl, pH 8.0) [15].

### ***High throughput screening of active compounds against M***^***pro***^

FRET screening was carried out for identifying the inhibitors of SARS-CoV-2 M^pro^. The fluorogenic substrate of M^pro^ (MCA-AVLQSGFRKK-Lys (Dnp) -Lys-NH_2_) was purchased from GL Biochem Ltd. (Shanghai, China). The screening system was established by employing M^pro^ (200 nM), substrate (20 µM), and the tested compounds with the different concentrations from 0.039 to 160 µM. The excitation and emission wavelength were set at 320 and 405 nm, separately. The data was analyzed with Graphpad prism 9.0 and all experiments were performed in triplicates [16].

### ***The ligand-binding affinity with M***^***pro***^

*SPR assay*. Biacore T200 system was used for SPR assay. M^pro^ was conjugated to a sensor Chip (CM5) via amino coupling reaction [17]. The tested compounds were diluted with the running buffer (1.05 × PBS-P, 5% DMSO) to reach the working concentration of 0.977–500 µM, being injected into the channels at a flow rate of 30 µL/min. The coupling time and the dissociation time were respectively set at 60 s and 120 s or 180 s. The data were analyzed for fitting the equilibrium dissociation constant (*K*_*D*_) [18, 19].

*ITC measurement*. ITC is the standard assay for measuring affinity interactions, which determines the thermodynamic parameters with label-free outputs [20]. The tested compounds and M^pro^ were dissolved by the same buffer at the suitable concentration, respectively. The cell filled with water as the reference, and the sample solution of the tested compound (500 µM) was titrated to the sample cell filled with M^pro^ (40 µM) in temporal sequence. The relevant parameters were monitored and documented, such as the thermodynamic equilibrium constant (*K*), enthalpy variations (*ΔH*), entropy variation (*ΔS*), and binding stoichiometry (*N*) [21].

### Cell culture and cytotoxicity assay

Vero E6 (ATCC, CRL-1586) cells (1 × 10^4^ cells/well) were maintained in a 96-well plate for 12 h. All cells were cultured in complete medium composed of Dulbecco’s Modified Eagle Medium (DMEM) (Gibco, USA), 10% (v/v) serum, 100 U/mL penicillin, and 100 µg/mL streptomycin. The diluted solutions of the tested compounds (200 µL) at the concentrations of 100, 80, 60, 40, 20, 10, and 5 µM were added to wells and incubated for another 24 or 48 h, respectively. At the terminus period of incubation, 50 µL MTT (2.5 mg/mL) was added to each well and continuously incubated at 37 ℃ for 4 h. After disposal of the liquid supernatant, 150 µL DMSO was added to each well, whose absorbance was detected at 490 nm via Microplate Reader (TECAN SunriseTM, China). Cytotoxicity was calculated as follows: Cell viability (%) = (OD_experiment_-OD_Blank_)/(OD_control_-OD_Blank_) × 100%. The data were analyzed with GraphPad Prism 9.0. All experiments were performed in triplicates.

### Antiviral activities assay

Vero E6 (ATCC, CRL-1586) cells were seeded in a 96-well plate at 2 × 10^4^ cells/well in a fully humidified cultivation chamber at 37 ℃ containing 5% CO_2_ and bacteria-free condition. Omicron BA.1.1 and Omicron BA.2.3 variants were respectively added and propagated in Vero E6 cells. After inoculation for 3 days, a cytopathic effect (CPE) was scored by Celigo Image Cytometer, and the Reed-Muench formula was used to calculate the 50% tissue culture infectious dose (TCID_50_) as the reference.

The solutions of the tested compound with the different concentrations were respectively mixed with Omicron BA.1.1 and BA.2.3 variants (MOI = 0.01). Then, 200 μL mixture was added to a monolayer Vero E6 cells. After incubation for 48 h, CPE was scored, from which the value of half-maximal effective concentration (EC_50_) and the antiviral activity of the tested compounds were evaluated [22]. All experiments were performed at BSL-3 level laboratory in Guangzhou Customs Inspection and Quarantine Technology Center (IQTC).

### Molecular docking

The crystal structure of SARS-CoV-2 M^pro^ (PDB ID: 6LU7) [23] and Omicron M^pro^ (PDB ID: 7TLL) was respectively downloaded from the Protein Data Bank database (http://www.rcsb.org/pdb/), which is acquired from the crystal complex structure of M^pro^-N3 and M^pro^-nirmatrelvir. The 3D structures of the tested natural compounds in our compounds library were downloaded from ChemSpider (https://www.chemspider.com/) and PubChem (https://pubchem.ncbi.nlm.nih.gov/) databases. The ligands and the proteins were prepared with Discovery Studio 2020. Energies minimization and the ensembles of ligands’ conformations were optimized. The structure of M^pro^ was prepared by removing the water molecules, adding hydrogen, fixing the missing residues or loops, and defining the binding site of original ligand as the active center. Then, the tested compounds were docked to M^pro^ by CDOCKER module [24], from which the CDOCKER interaction energy score was ranked to predict the interaction capacity.

### Molecular dynamics (MD) simulation

MD simulation was performed with GROMACS 2018. The topologies of M^pro^ and ligands were prepared based on the CHARMM36 force field and the ChARMM-GUI server, respectively [25]. The complexes were solvated via the TIP3P water model in a dodecahedral periodic box with nearly 2.5 nm in all directions, which was balanced in the intracellular environment by adding 0.15 M NaCl. The 50,000 steps of steepest descent algorithm were used for minimizing energy [26]. The canonical ensemble, constant-pressure, and constant-temperature equilibration simulation in systems were carried out about 300 ns. The electrostatic interactions were described using the particle mesh Ewald (PME) algorithm with a cutoff of 1.2 nm. The LINear Constraint Solver (LINCS) algorithm [27] was used to constrain the bonds. The system pressure was maintained semi-isotopically at 1 bar in the x, y, and z-directions via the Berendsen barostat. The system temperature was maintained at 300 K via the Berendsen thermostat. The time of MD simulation was set at 2 fs. The analysis was carried out by the Gromacs software package, including GMX RMS, GMX RMSF, GMX gyration, GMX COVAR, and GMX ANAeig modules [28].

### Analysis of NMR spectra

NMR spectra were employed to demonstrate the formation of hydrogen bond between the tested compound and M^pro^, including saturation transfer difference (STD) and water-ligand observed via gradient spectroscopy (WaterLOGSY). The tested compound (acteoside) and M^pro^ were respectively dissolved in D_2_O, which were employed to prepare the sample solutions for STD analysis (C_acteoside_: C_Mpro_ at about 100: 1) and WaterLOGSY analysis (C_acteoside_: C_Mpro_ at about 10: 1) [29]. The spectra were recorded by 256 scans with 16 ppm spectral width at 600 MHz. The temperature was fixed at 298 K. The data were analyzed by mestrenova software (Version 9.0).

## Results

### ***High throughput screening of M***^***pro***^*** inhibitors from XFBD by FRET assay***

**Fig. 2:**
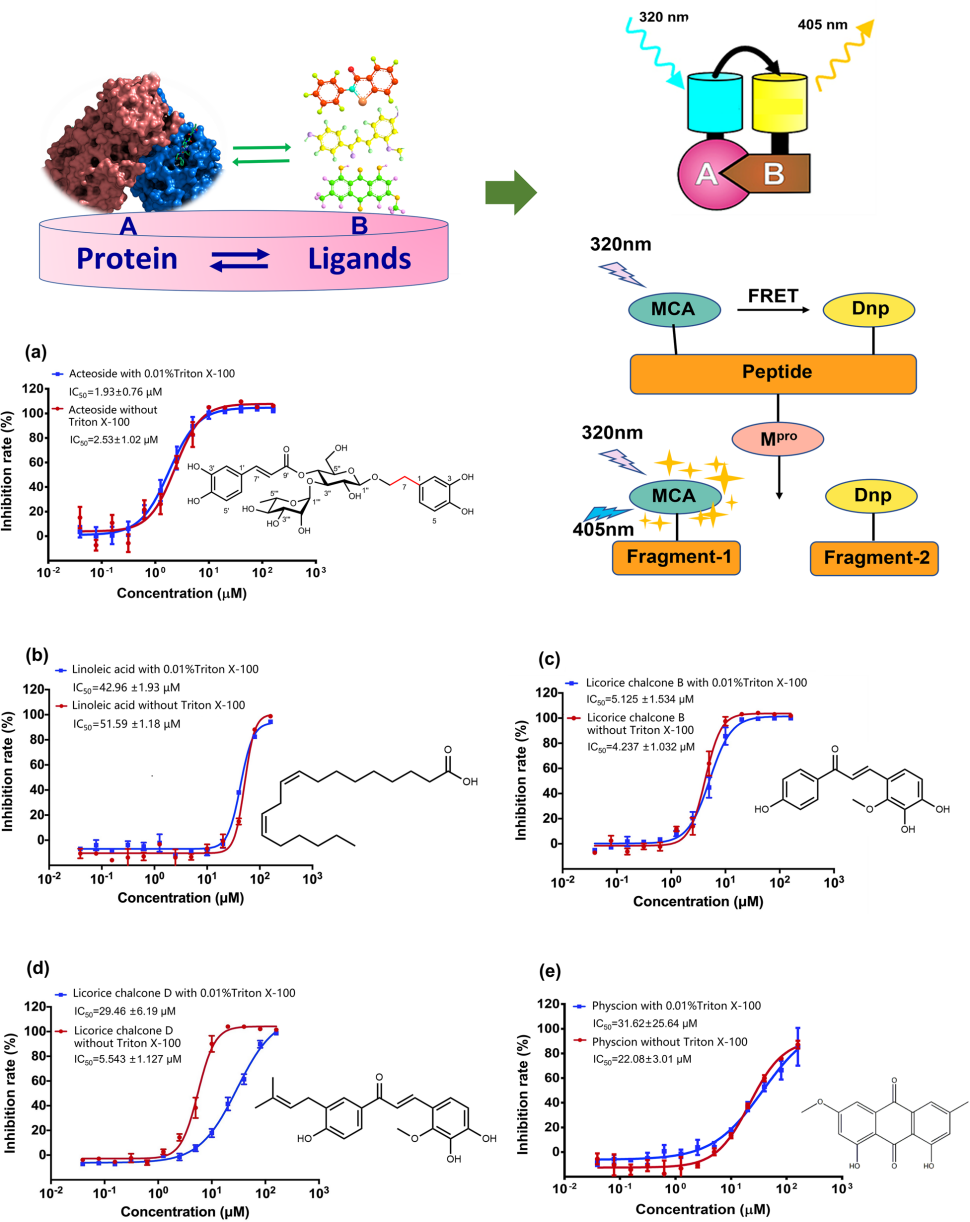
Inhibition activity of the tested compounds on SARS-CoV-2 M^pro^ (n = 5)

As a high throughput screening method, FRET is frequently employed to identify the potentially active compounds by measuring the interaction between the tested compounds and target proteins. By employing M^pro^ with purity above 95% purified in our laboratory (Additional file 1: Fig. S6), a total of 83 natural compounds were screened by FRET to identify the potential active compounds in XFBD. Linoleic acid, acteoside, licochalcone D, licochalcone B, and physcion exhibited satisfactory activities with the inhibition rate above 90% at 40 µM (Additional file 1: Fig. S7), which showed M^pro^ inhibition activity with IC_50_ ranging from 1.93 to 42.96 µM (Fig. 2). It was reported that up to 19% of small molecules preferentially form aggregates at 30 µM, which could nonspecifically inhibit the protease activity [30]. As a detergent, triton X-100 was added to the tested system to reach 0.01% (*m: v*) for preventing the molecule aggregation. Interestingly, licochalcone D obviously fluctuated in IC_50_. When without triton X-100, the inhibition activity of licochalcone D was drastically elevated from 29.46 to 5.54 µM (Fig. 2d), which possibly derived from the nonspecific inhibition on M^pro^ caused by self-aggregation of licochalcone D, resulting in the diverged inhibition activity on M^pro^ [31]. Therefore, acteoside and licochalcone B were suggested as the potential M^pro^ inhibitors.

### ***The binding affinities between the tested compounds and M***^***pro***^*** determined by SPR***

SPR is employed to measure the ligand-M^pro^ interaction in real-time, which is widely accepted in determination of the binding affinity, including dissociation rate constant (*K*_*d*_), association rate constant (*K*_*a*_), and dissociation constant (*K*_*D*_*, K*_*D*_ = *K*_*d*_*/K*_*a*_).

**Fig. 3:**
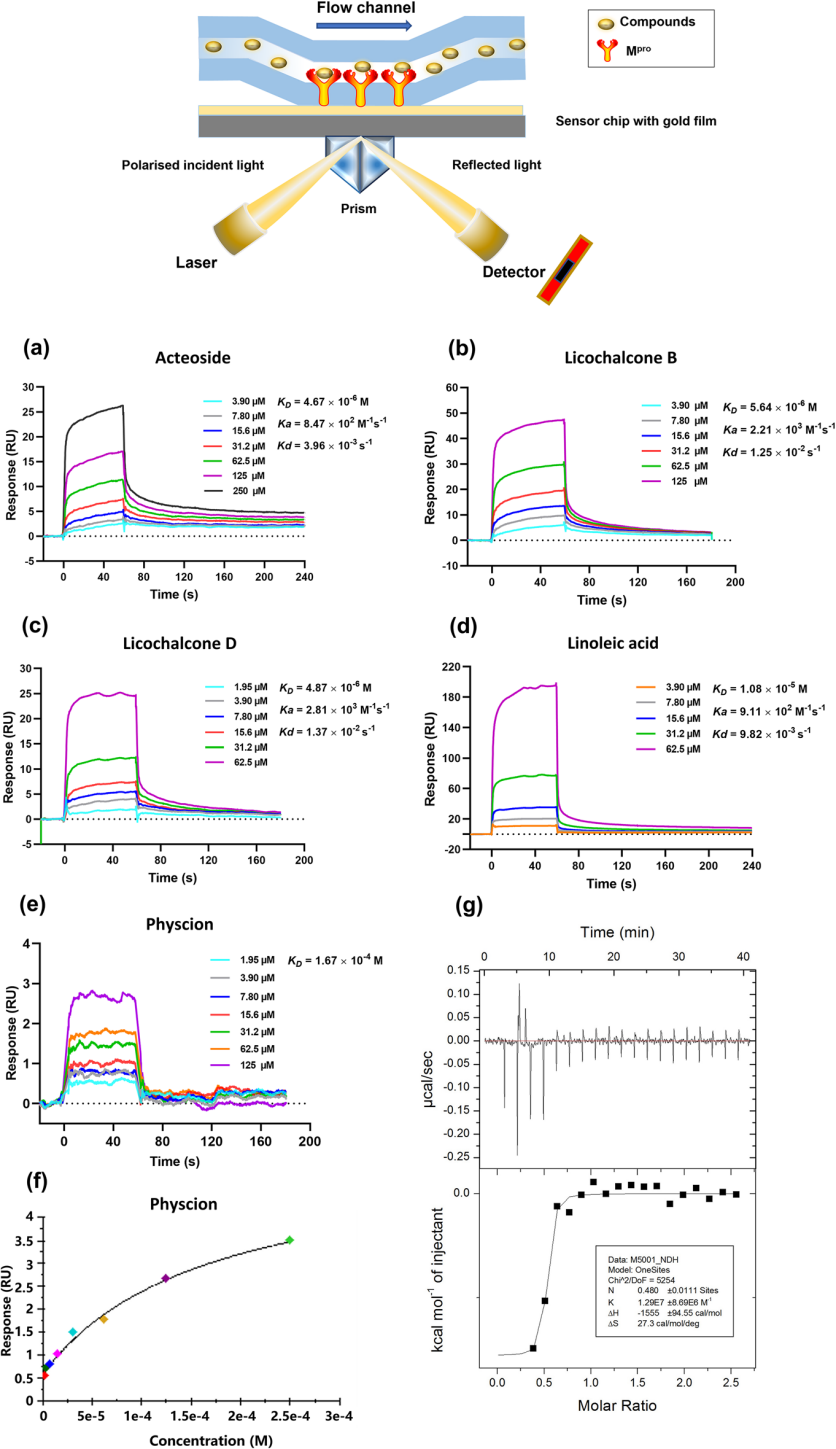
The SPR affinity curves of acteoside (**a**), licochalcone B (**b**), licochalcone D (**c**) and linoleic acid (**d**) fitted by kinetic mode, and physcion fitted by affinity mode (**e** and **f**). The calorimetric titration curve of acteoside-M^pro^ complex at 298 K (**g**)

In our study, the five focused compounds by FRET were passed over the sensor’s surface to evaluate their affinities to M^pro^. The binding responses in resonance units (RU) were continuously recorded. As shown in Fig. 3a–f, the tested compounds showed the increased SPR response in a dose-dependent manner. Acteoside, licochalcone B, and licochalcone D showed good binding affinities with *K*_*D*_ values from 4.67 to 5.64 µM. Linoleic acid showed a moderate binding affinity with *K*_*D*_ value at 10.8 µM. Physcion exhibited a weak response with *K*_*D*_ value at 167 µM. In general, acteoside and licochalcone B were employed as potential active compounds by taking the screening results of FRET and SPR into consideration.

With superior solubility, acteoside was further studied by ITC to obtain the thermodynamic signatures with the thermodynamic equilibrium constant (*K*), enthalpy variations (*ΔH*), entropy variations (*ΔS*), and binding stoichiometry (*N*) [32]. The calorimetric titration profiles of acteoside to M^pro^ was exhibited in Fig. 3g. The* K* of acteoside bound with M^pro^ was 0.129 µM at 298 K, *ΔH* was − 1.55 ± 0.094 kcal/mol, and *ΔS* was 27.3 cal/mol/deg. Negative values of *ΔH* and positive value of *ΔS* suggested that the binding of acteoside with M^pro^ was preferentially driven by *ΔH*, which displayed the electrostatic attraction and hydrophobic interaction as the key modes of interaction in the formation of acteoside-M^pro^ complex [33, 34]. The *N* value shows 0.48, indicating that acteoside binds to M^pro^ complex with a 1:0.48 stoichiometry, which possibly cause M^pro^ dimerization [19].

### *Antiviral activities on Omicron BA.1.1 and BA.2.3 variants *in vitro

**Fig. 4:**
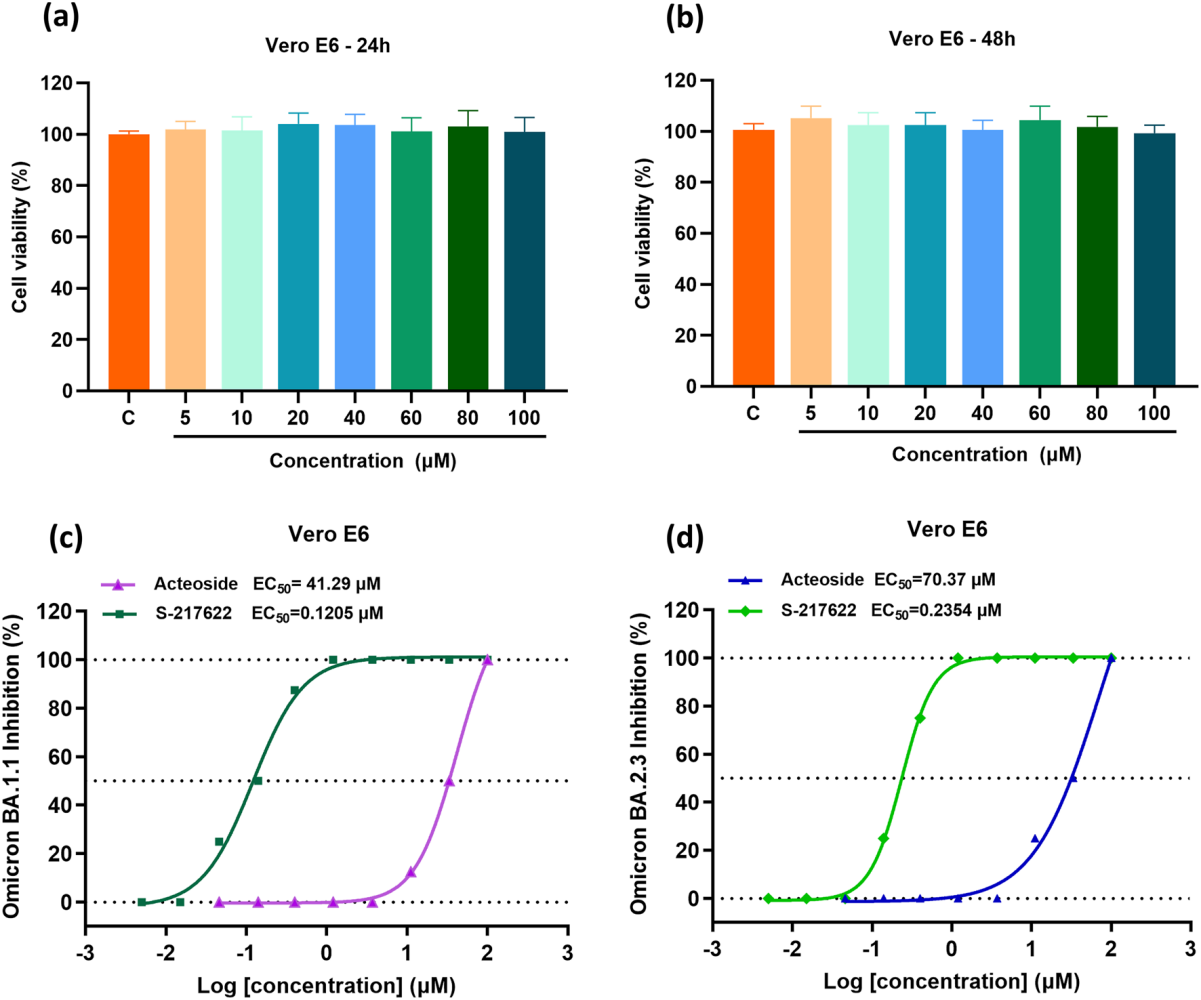
Cytotoxicity studies of acteoside by MTT assay (**a** and **b**) and antiviral activities on Omicron BA.1.1 and BA.2.3 variants in Vero E6 cells (**c** and **d**)

By MTT assay, acteoside showed no inhibitive effect on Vero E6 cells proliferation up to 100 µM for 24 or 48 h, which gave the half-cytotoxic concentration (CC_50_) > 100 µM (Fig. 4a and b). In antiviral activities assay, S-217622 was applied as a positive control to evaluate the reliability of the experimental system, which is the first oral noncovalent and nonpeptidic SARS-CoV-2 M^pro^ inhibitor as a clinical candidate [35]. The positive drug and the tested compound solutions with the different concentrations separately mixed with virus were added to the medium of Vero E6 cells for infection in 48 h, respectively. Then, the CPE score was used to evaluate antiviral activity by determining EC_50_ of acteoside. As shown in Fig. 4c and d, EC_50_ values of positive drug was 0.1205 μM on Omicron BA.1.1 and 0.2354 μM on Omicron BA.2.3. And EC_50_ values of acteoside were 41.29 µM for Omicron BA.1.1 and 70.37 µM for BA.2.3. As a focused active compound in XFBD, acteoside exhibited promising inhibitory effects against both Omicron BA.1.1 and BA.2.3 variants via inhibition of M^pro^ activity, which is consistent with the reported inhibition activity on SARS-CoV-2 M^pro^ by *in-silico* approach [36]. Given that the dominant spread of Omicron variants BA.1.1 and BA.2.3, the antiviral activity of acteoside may be meaningful in the clinical application for treating COVID-19 patients. Also, as a main compound from *verbenae herba* in XFBD, acteoside performs anti-inflammatory, anti-oxidant, cardiovascular protective, and immunoregulation [37], conducing to the recovery of COVID-19 patients.

### Unveilment of the antivirus mechanism for acteoside by in-silico approach

**Fig. 5:**
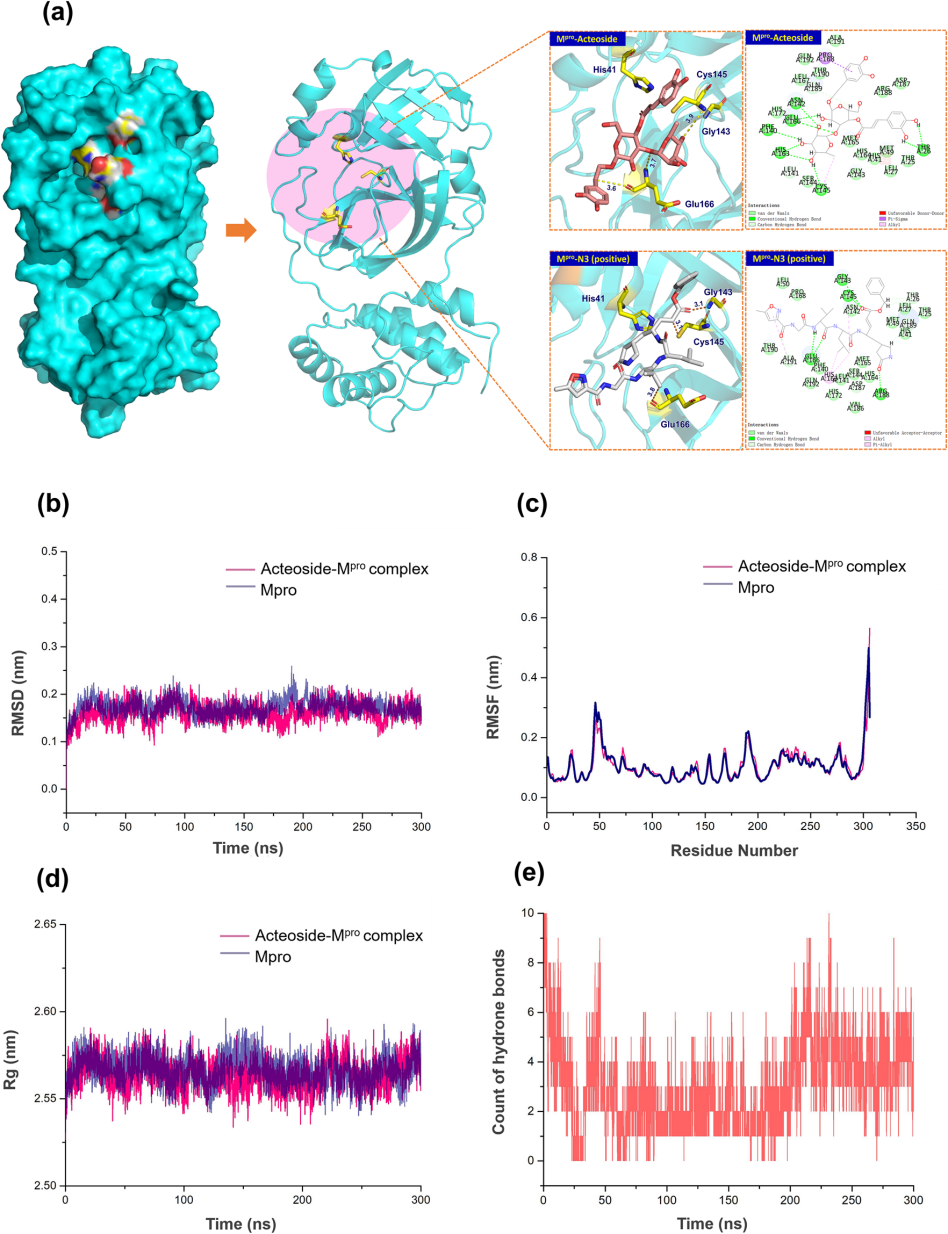
The predicted structure of the acteoside-M^pro^ and N3-M^pro^ complexes (**a**) (M^pro^ is colored in blue, N3 is colored in grey and acteoside is colored in salmon). The results of RMSD (**b**), RMSF (**c**) and Rg analysis (**d**), and count of hydrogen bonds between acteoside and M^pro^ (**e**)

A homodimer is regarded as the functional unit of M^pro^ composed of two monomers. Domain I, domain II, and domain III are the three major domains of M^pro^. The Cys145-His41 catalytic dyad between domain I and domain II is set as the active site. Based on the model of the crystal complex structure of SARS-CoV-2 M^pro^-N3, we docked acteoside into the active pocket to estimate the binding modes by the CDOCKER interaction conformation with minimum binding free energy. As shown in Fig. 5a, acteoside and N3 (representation in stick) are respectively aligned in the pocket of M^pro^, highlighting their location and distance to the residues. One conformation of acteoside fits perfectly in the binding pocket of M^pro^, which is similar to N3. The CDOCKER interaction energy of N3-M^pro^ is − 72.88 kcal/mol and acteoside-M^pro^ is − 68.78 kcal/mol. By docking acteoside to M^pro^, it showed that hydrogen bonds were formed with Cys145^3.9^ and Glu166^3.6^ as side chain residues of M^pro^, which is essential for exerting its inhibition activity.

According to the results of root mean square deviation (RMSD) and root mean square fluctuation (RMSF) analysis (Fig. 5b and c), the stability of acteoside-M^pro^ complex was predicted to be satisfactory with the minor deviation in RMSD and the stable intermolecular interactions in the simulated trajectory [38]. As shown in Fig. 5d, close to M^pro^, the radius of gyration (Rg) of acteoside-M^pro^ complex shows a better compactness, indicating the perfect interaction between acteoside and M^pro^. Hydrogen bonds between acteoside and residues of M^pro^ were also observed at the early phase of the simulation and tended to be stable during the entire simulation period, which were considered as the primary interaction mode between acteoside and M^pro^ [39].

### ***Uncovering the intermolecular interaction between acteoside and M***^***pro***^*** by NMR spectroscopy***

**Fig.6:**
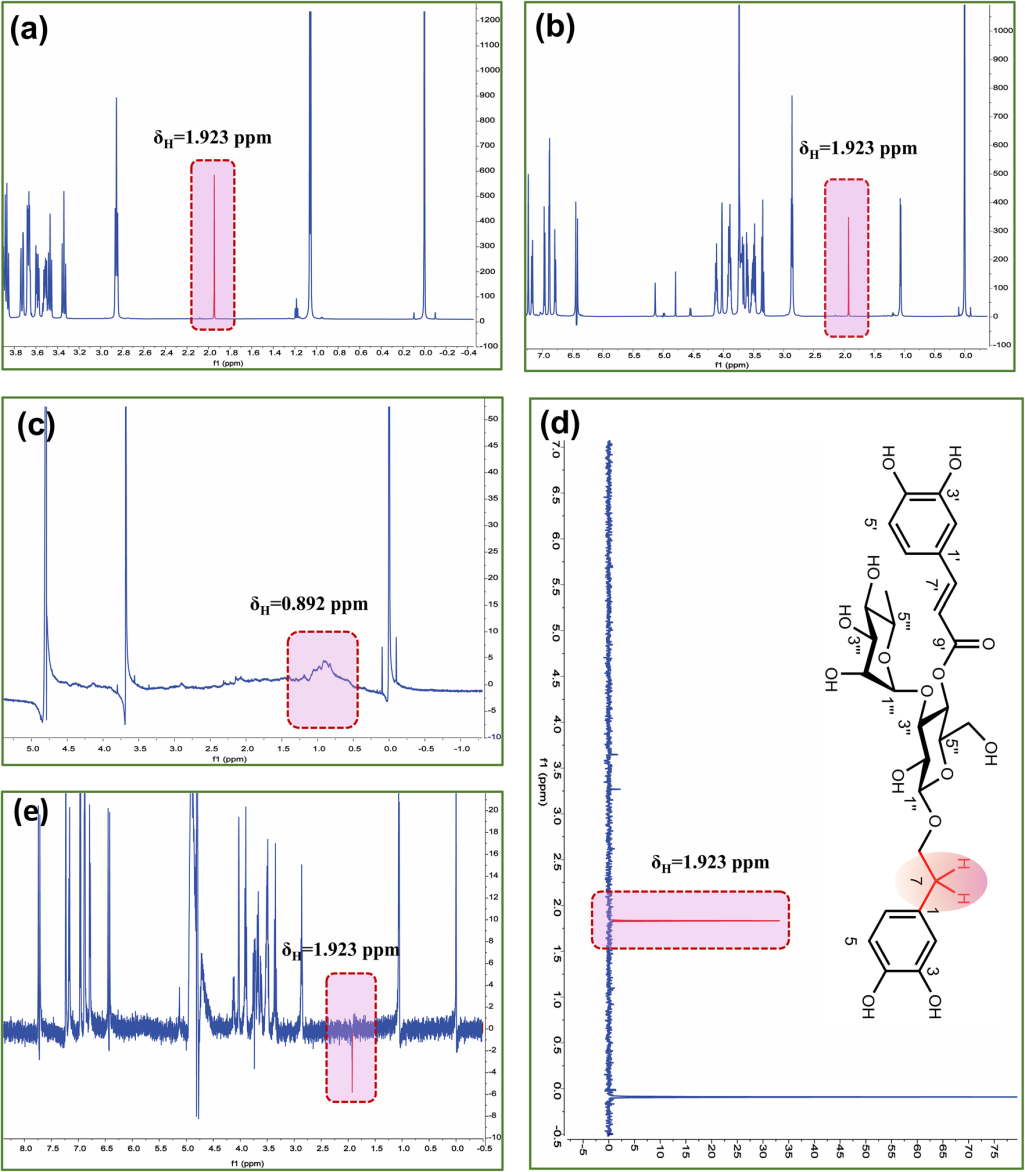
^1^H NMR spectra of acteoside (**a**), acteoside-M^pro^ complex (**b**), M^pro^ (**c**), STD (**d**), and WaterLOGSY NMR spectra of acteoside-M^pro^ complex (**e**)

Based on the results of the molecular docking and molecular dynamic simulations, the STD and WaterLOGSY NMR spectra are employed to identify the ligand binding epitopes. In the STD NMR testing, the identified hydrogen receives the most intense magnetization transfer [40], which is suggested as the most intimate contact between the tested compound and protein [41, 42]. Presented in the Fig. 6 are the ^1^H NMR spectra of acteoside (a), acteoside-M^pro^ complex (b), M^pro^ (c), and STD (d). The STD NMR analysis showed that acteoside was bound to M^pro^ by H-atom at δ_H_ = 1.923 ppm, which was identified at *7-H* of acteoside.

WaterLOGSY NMR spectrum also based on magnetization transfer, which is another method for evaluating the binding affinity between acteoside and M^pro^ to evidently support the STD NMR results. In WaterLOGSY NMR spectrum, the detected signals represents the transfer of magnetization among water molecules, proteins, and the tested compound via the nuclear overhauser effect (NOE) and chemical exchange [43]. As the rotational correlation time increases, the signal of the interacted hydrogens with the target protein from the tested compound turns to be negative. As shown in Fig. 6e, the signal is found to be negative at δ_H_ = 1.923 ppm, suggesting that the H-bond interaction occurs between acteoside and M^pro^ by *7-H*, which is in line with result of STD. Accordingly, acteoside inhibit M^pro^ activity via the formation of hydrogen bond at *7-H* of acteoside.

## Discussion

Since the outbreak of COVID-19 in December 2019, it caused the number of infections and enormous pressure of intensive care for medical organizations. Lots of conventional antiviral drugs were used for screening the specific anti-SARS-CoV-2 candidates. To our disappointment, multiple drugs show the poor antiviral effects. Until now, molnupiravir and paxlovid (PF-07321332) are the two oral anti-SARS-CoV-2 drugs, which has been approved for marketing via FDA. However, these two drugs are not suitable for universal drugs for treating COVID-19 patients due to occurrence of high-risk groups by its side effects or drug resistance [41]. The vaccines, as the first line against the virus, exhibited the unsatisfactorily protective effect when patients were exposed to mutant strains, whose safety and efficacy remain to be elucidated. Whether fighting against SARS-CoV-2 or Omicron strains, the TCMs show an evidently therapeutic effect, which may be a gold mine for antiviral drugs discovery [44, 45]. XFBD is recommended for the treatment of COVID-19 in China and also approved in Canada, Uzbekistan, and so on. However, the antiviral mechanisms are less understood.

**Fig. 7:**
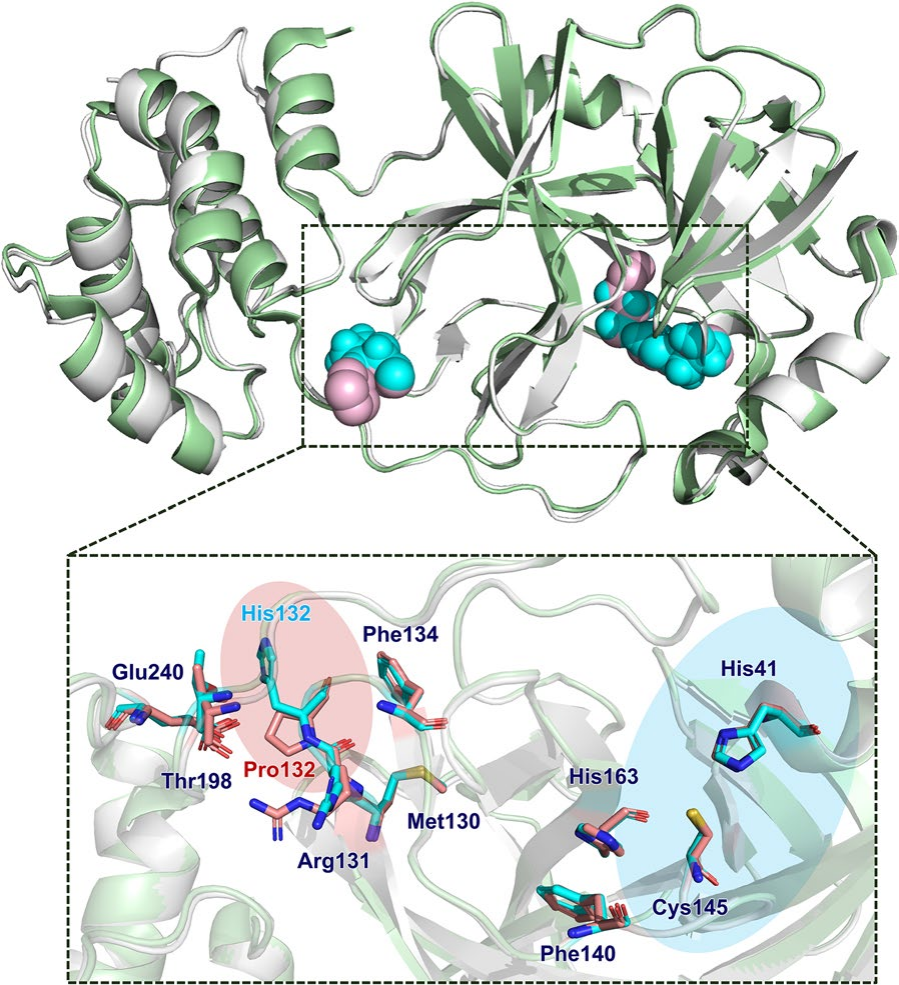
The X-ray crystal structure of WT M^pro^ (SARS-CoV-2 M^pro^, PDB ID: 6LU7, *gray*) and Omicron M^pro^ (SARS-CoV-2 M^pro^ P132H, PDB ID: 7TLL, *green*). The residues of Omicron M^pro^ (*magenta*) superposed on the WT M^pro^ (*blue*)

M^pro^ is a well-known target for drug discovery and designing, which plays the indispensable role in the replication of SARS-CoV-2 [46]. Focusing on M^pro^, we screened and evaluated a series of compounds from XFBD to identify the antiviral bioactive compounds and clarify the potential mechanism by means of virtual screening and experimental verification. In the end, acteoside, as a representative active compound, was confirmed to be an effective inhibitor on wild-type M^pro^ (WT M^pro^). The substrate binding pocket on SARS-CoV-2 M^pro^ is highly conserved among all coronaviruses. Compared to WT M^pro^ His132, mutation in Pro132 is observed in M^pro^ P132H of Omicron variants, which decreases thermal stability without compromising catalysis or small molecule drugs inhibition [47]. As shown in Fig. 7, P132H doesn’t change the conformation of the Cys145-His41 catalytic pocket, which shows the comparable catalytic activity compared to WT M^pro^ His132 [48]. In order to identify the binding form, the complex of nirmatrelvir-M^pro^ P132H (PDB ID: 7TLL) was employed to dock acteoside to Omicron M^pro^ (Additional file 1: Fig. S8). The CDOCKER interaction energy is − 65.44 kcal/mol for nirmatrelvir-M^pro^ P132H and − 62.40 kcal/mol for acteoside-M^pro^ P132H, showing the excellent activity of acteoside against Omicron M^pro^. Moreover, the limitation of observable differences was observed in conformation between acteoside-WT M^pro^ and acteoside-Omicron M^pro^. Therefore, whatever WT M^pro^ or Omicron M^pro^, they could be employed as the screening target to identify antiviral candidates. With the continuous mutation of SARS-CoV-2, WT strain is rarely popular and has been instead of Omicron strains. Omicron variants are more invasive to immune system, resulting in higher transmissibility. Amazingly, acteoside inhibited omicron BA.1.1 and BA.2.3 in a dose-dependent manner.

Acteoside is a phenylpropanoid glycoside, which was identified in XFBD via high-resolution MS (2021) [49]. In previous study, it was proved to enhance IFN-γ and then selectively activate T cells in mouse lymphocytes [50]. In vitro, acteoside has been demonstrated to have antivesicular stomatitis virus (VSV) activity. Moreover, acteoside can effectively inhibit influenza infection in vivo. As a natural product, acteoside is observed to be safe up to 800 mg/kg in mice [51]. Our finding provides a new avenue for antiviral applications of acteoside through inhibiting M^pro^ activity to block the replication of SARS-CoV-2.

## Conclusions

As a representative compound, acteoside showed excellent inhibition on M^pro^ activity by the intermolecular interaction. Acteoside was successfully proved to display anti-Omicron BA.1.1 and anti-Omicron BA.2.3 activities in vitro. By computational modeling and NMR testing, we unveiled the intermolecular hydrogen bond formed by *7-H* of acteoside and M^pro^, resulting in inhibition of M^pro^ activity, which was speculated to be the antiviral mechanism of acteoside. In general, our study provides the strategy and procedures for rapid and effective identification of antiviral compounds and elucidation of its mechanism, conducing to scientifical application of formula in treating COVID-19 patients.

## Supplementary Information

**Additional file 1: Table S1**. The composition of XFBD. **Fig S1.** Analysis of network pharmacology of XFBD against COVID-19. (a) The herbs-components-targets network; (b) The herbs-components-core targets-disease network; (c) The targets-pathways network; (d) Bubble plots and (e) histogram for GO enrichment analysis; (f) Bubble plots and (g) histogram for KEGG enrichment analysis. **Fig S2.** The pathways of COVID-19 infection and the key targets marked as a red square for XFBD in treatment of COVID-19. **Fig S3.** The intersection of active compounds targets and disease targets by Venn diagram. **Fig S4.** The network of protein-protein interaction (PPI). **Fig S5.** Degree values of targets. **Table S2**. The informations of the tested compounds. **Fig S6.** The purified Mpro with the purity above 95%. **Fig S7.** The inhibition activity of the tested compounds on Mpro at 40 µM. **Fig S8.** The predicted structure of the acteoside-Mpro P132H and Nirmatrelvir-Mpro P132H complexes. WT Mpro His132 (gray and blue) and Mpro P132H (green and pink) are superposed. **Fig S9.** 1H NMR spectrum of acteoside

## Data Availability

All data used to support the claims of the study can be found in the main manuscript and supplemental materials. Raw data is available from the corresponding author upon reasonable request.

## Abbreviations

COVID-19: Coronavirus disease 2019
M^pro^: Main protease
PDB: Protein data bank
SARS-CoV-2: Severe acute respiratory syndrome coronavirus 2
SPR: Surface plasmon resonance
TCM: Traditional Chinese medicine
XFBD: Xuanfei Baidu formula
WHO: The World Health Organization
FRET: Fluorescence resonance energy transfer
ITC: Isothermal titration calorimetry
NMR: Nuclear magnetic resonance
MD: Molecular dynamics
TCID_50_: 50% tissue culture infectious dose
STD: Saturation transfer difference
NMPA: National Medical Products Administration
Rg: Radius of gyration
RMSD: Root mean square deviation
RMSF: Root mean square fluctuation
CC_50_: Half-cytotoxic concentration
CPE: Cytopathic effect
EC_50_: Half-maximal effective concentration
IC_50_: Half-maximal inhibitory concentration
MOI: Multiplicity of infection
