# Supplementary material for: Discovery and evaluation of active compounds from Xuanfei Baidu formula against COVID-19 via SARS-CoV-2 Mpro
Source: Chin Med. 2023 Aug 2;18:94. doi: 10.1186/s13020-023-00790-0 (PMC10394814; doi:10.1186/s13020-023-00790-0)
Supplement: Supplementary file 1 — Additional file 1: Table S1. The composition of XFBD. Fig S1. Analysis of network pharmacology of XFBD against COVID-19. (a) The herbs-components-targets network; (b) The herbs-components-core targets-disease network; (c) The targets-pathways network; (d) Bubble plots and (e) histogram for GO enrichment analysis; (f) Bubble plots and (g) histogram for KEGG enrichment analysis. Fig S2. The pathways of COVID-19 infection and the key targets marked as a red square for XFBD in treatment of COVID-19. Fig S3. The intersection of active compounds targets and disease targets by Venn diagram. Fig S4. The network of protein-protein interaction (PPI). Fig S5. Degree values of targets. Table S2. The informations of the tested compounds. Fig S6. The purified Mpro with the purity above 95%. Fig S7. The inhibition activity of the tested compounds on Mpro at 40 µM. Fig S8. The predicted structure of the acteoside-Mpro P132H and Nirmatrelvir-Mpro P132H complexes. WT Mpro His132 (gray and blue) and Mpro P132H (green and pink) are superposed. Fig S9. 1H NMR spectrum of acteoside [file 13020_2023_790_MOESM1_ESM.docx]

***Supplementary Material***

**Discovery and evaluation of active compounds from Xuanfei Baidu formula against COVID-19 via SARS-Cov-2 M^pro^**

**Min Zhang^1,4,5, †^, Liting Liu^1, †^, Yao Zhao^6^, Yipeng Cao^7^, Yan Zhu^6^, Lifeng Han^1,3,5^, Qi Yang^8^, Yu Wang^1,4^, Changjian Wang^1^, Han Zhang^1,4,5, *^, Yuefei Wang^1,3,5, *^, Junhua Zhang^1,2,5, *^**

^1^ State Key Laboratory of Component-Based Chinese Medicine, Tianjin University of Traditional Chinese Medicine, Tianjin 301617, China

^2^ Evidence-Based Medicine Center, Tianjin University of Traditional Chinese Medicine, Tianjin 301617, China

^3^ Tianjin Key Laboratory of TCM Chemistry and Analysis, Tianjin University of Traditional Chinese Medicine, Tianjin 301617, China

^4^ Key Laboratory of Pharmacology of Traditional Chinese Medical Formulae (Ministry of Education), Tianjin University of Traditional Chinese Medicine, Tianjin 301617, China

^5^ Haihe Laboratory of Modern Chinese Medicine, Tianjin 301617, China

^6^ Shanghai Institute for Advanced Immunochemical Studies and School of Life Science and Technology, ShanghaiTech University, Shanghai, 200031, China

^7^ National Supercomputer Center in Tianjin, Tianjin, 300457, China

^8^ Guangzhou Laboratory, Guangzhou 510005, China

***** Correspondence: zhanghan0023@126.com (H. Zhang), wangyf0622@tjutcm.edu.cn (Y. Wang), zjhtcm@foxmail.com (J. Zhang).

Tel: +86-22-59596366, Address: Tianjin University of Traditional Chinese Medicine, 10 Poyanghu Road, West Area, Tuanbo New Town, Jinghai District, Tianjin 301617, PR China.

^†^ These authors contributed equally to this work.

**1 The herbs of Xuanfei Baidu formula**

Xuanfei Baidu formula (XFBD), which derived from the classical formulae, consists of 13 herbs, as shown in table S1.

**Table S1 The composition of XFBD**

| Number | Latin name | English name | Chinese name | Abbreviation |
| --- | --- | --- | --- | --- |
| 1 | *Ephedrae Herba* | Ephedra | Shengmahuang | SMH |
| 2 | *Armeniacae Semen Amarnm* | Bitter Apricot Seed | Kuxingren | KXR |
| 3 | *Gypsum Fibrosum* | Gypsum | Shengshigao | SSG |
| 4 | *Coicis Semen* | Coix Seed | Shengyiyiren | SYYR |
| 5 | *Atractylodis Rhizoma* | Rhizoma Atractylodis | Maocangzhu | MCZ |
| 6 | *Pogostemonis Herba* | Cablin Patchouli Herb | Guanghuoxiang | GHX |
| 7 | *Artemisiae Annuae Herba* | Sweet Wormwood Herb | Qinghaocao | QHC |
| 8 | *Polygoni Cuspidati Rhizoma et Radix* | Giant Knotweed Rhizome | Huzhang | HZ |
| 9 | *Verbenae Herba* | European Verbena Herb | Mabiancao | MBC |
| 10 | *Imperatae Rhizoma* | Lalang Grass Rhizome | Ganmaogen | GMG |
| 11 | *Descurainiae Eemen Lepidii Semen* | Pepperweed Seed, Tansymustard Seed | Tinglizi | TLZ |
| 12 | *Citri Grandis Exocarpium* | Tomentose Pummelo Peel | Huajuhong | HJH |
| 13 | *Glycyrrhizae Radix et Rhizoma* | Licorice Roots Northwest Origin | Shenggancao | SGC |

**2 Prediction of the potential compounds in XFBD against COVID-19 by network pharmacology**

Except for gypsum, the components of 12 TCM herbs in XFBD were collected from traditional Chinese medicine systems pharmacology database (TCMSP, <https://old.tcmsp-e.com/tcmsp.php>). The protein was retrieved from the Uniprot database (https://www.uniprot.org/). The targets of COVID-19 were obtained from the human genecards database (https://www.genecards.org/). The network of herbs-components-targets-disease and the protein-protein interaction (PPI) network were visualized by the software cytoscape 3.8.1. By gene ontology (GO) and kyoto encydopedia of genes and genomes (KEGG) analysis, the effective clustering of functional can be obtained.

A total of 186 compounds in XFBD were screened out with oral availability (OB) ≥30% and drug-likeness (DL) ≥0.18. As shown in Fig. S1 a, the network of herbs-components-targets displayed 413 nodes and 2161 edges. In Fig. S1 b and c, VEGFA, CASP3, IL6, MAPK3, STAT3, MAPK1 may be the key targets of XFBD for the treatment of COVID-19. The analysis of GO and KEGG enrichment (Fig. S1 d ~ g) showed that the pathways of inflammation, and viral transcription, replication and translation are involved in treatment of COVID-19 by XFBD, including cytokine receptor binding, RNA polymerase II-specific DNA-binding transcription factor binding, and DNA-binding transcription factor binding.

The intersection of active compounds targets and disease targets shared 57 targets (Fig. S3), which were employed to construct the herbs-components-targets-disease network and the protein-protein interaction (PPI) network (Fig. S4). As shown in Fig. S5, degree values of targets were displayed, from which VEGFA, CASP3, IL-6, MAPK3, and STAT3 were the potential core targets to participate in efficacy response.

Base on the results of network pharmacology, we built the compounds library including 83 compounds (Table S6), which are available in the market.


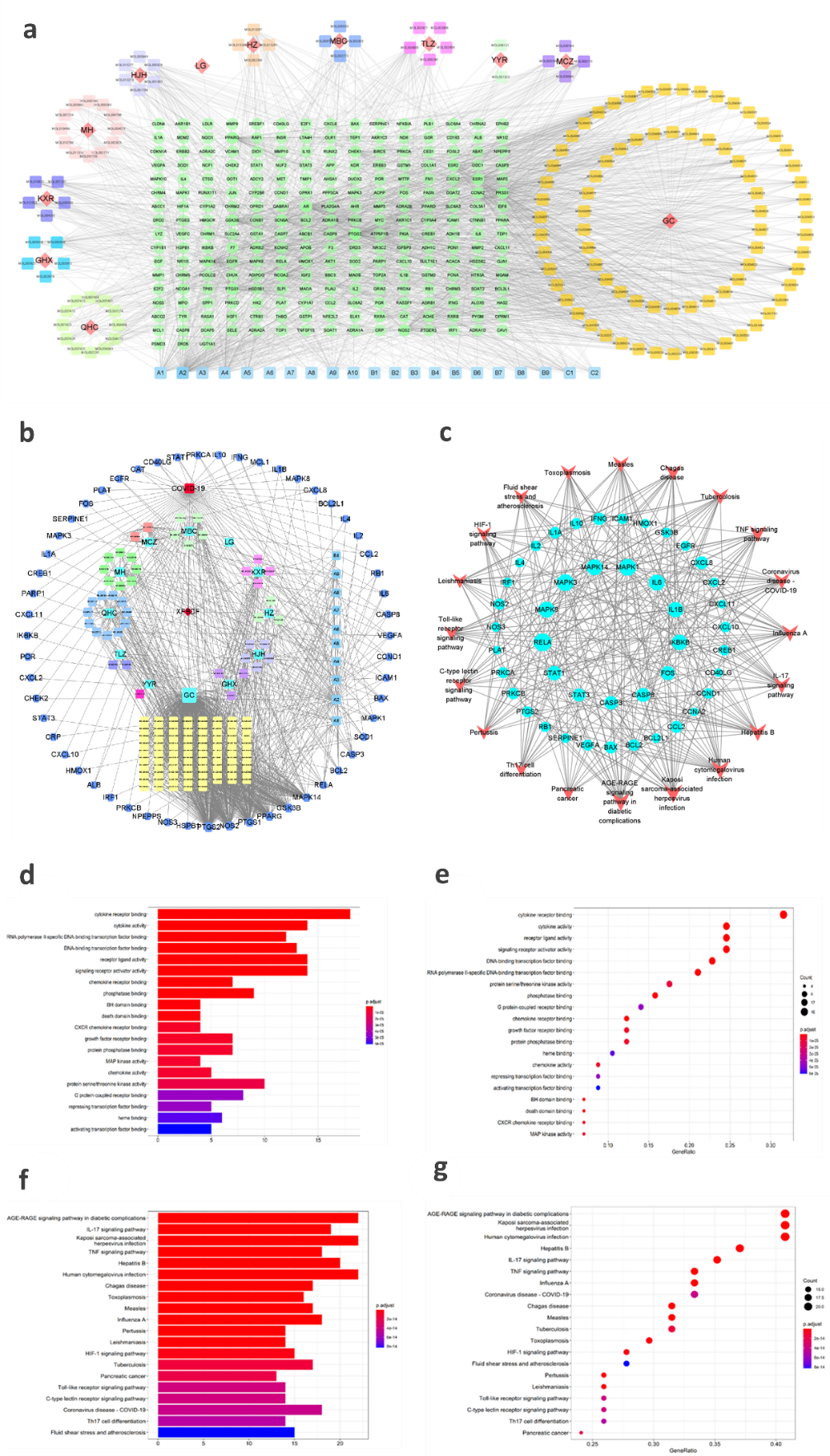


**Fig. S1****.** Analysis of network pharmacology of XFBD against COVID-19. (a) The herbs-components-targets network; (b) The herbs-components-core targets-disease network; (c) The targets-pathways network; (d) Bubble plots and (e) histogram for GO enrichment analysis; (f) Bubble plots and (g) histogram for KEGG enrichment analysis.


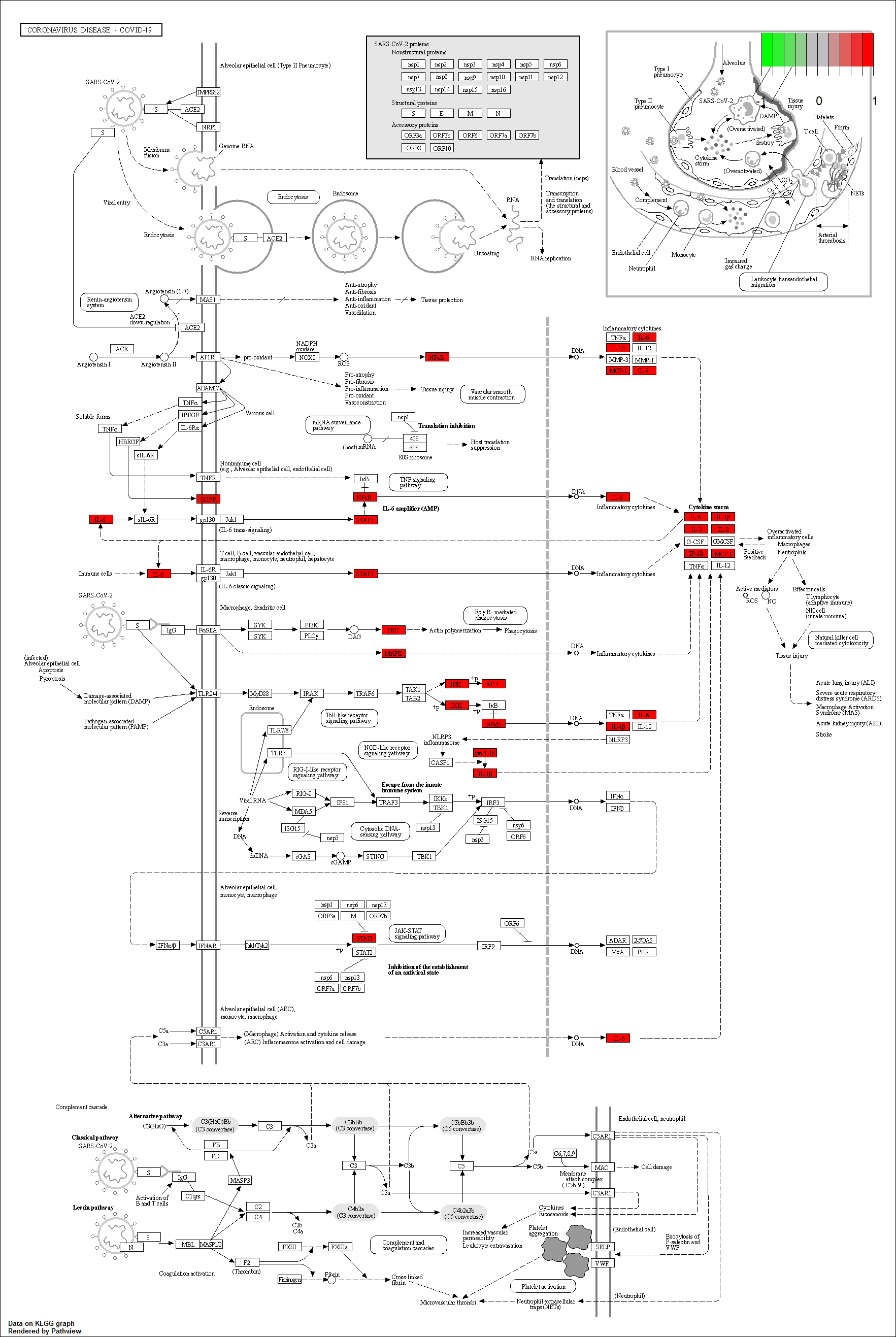


**Fig. S2.** The pathways of COVID-19 infection and the key targets marked as a red square for XFBD in treatment of COVID-19.


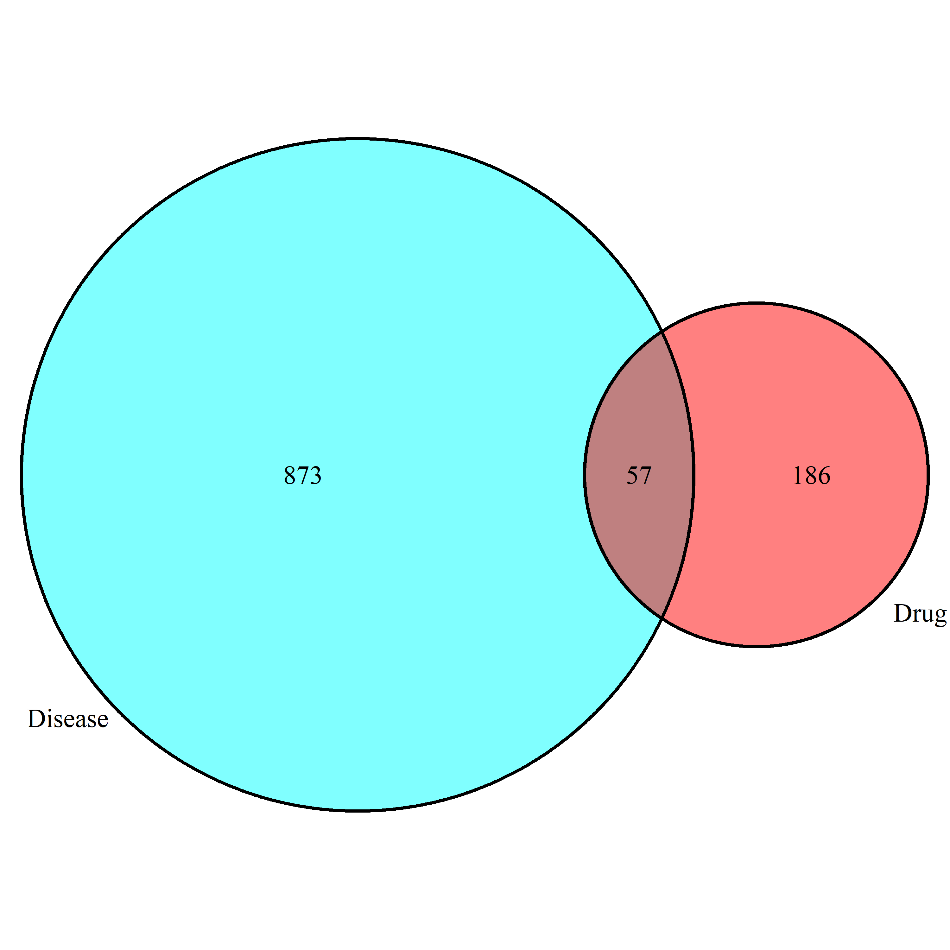


Fig. S3. The intersection of active compounds targets and disease targets by Venn diagram
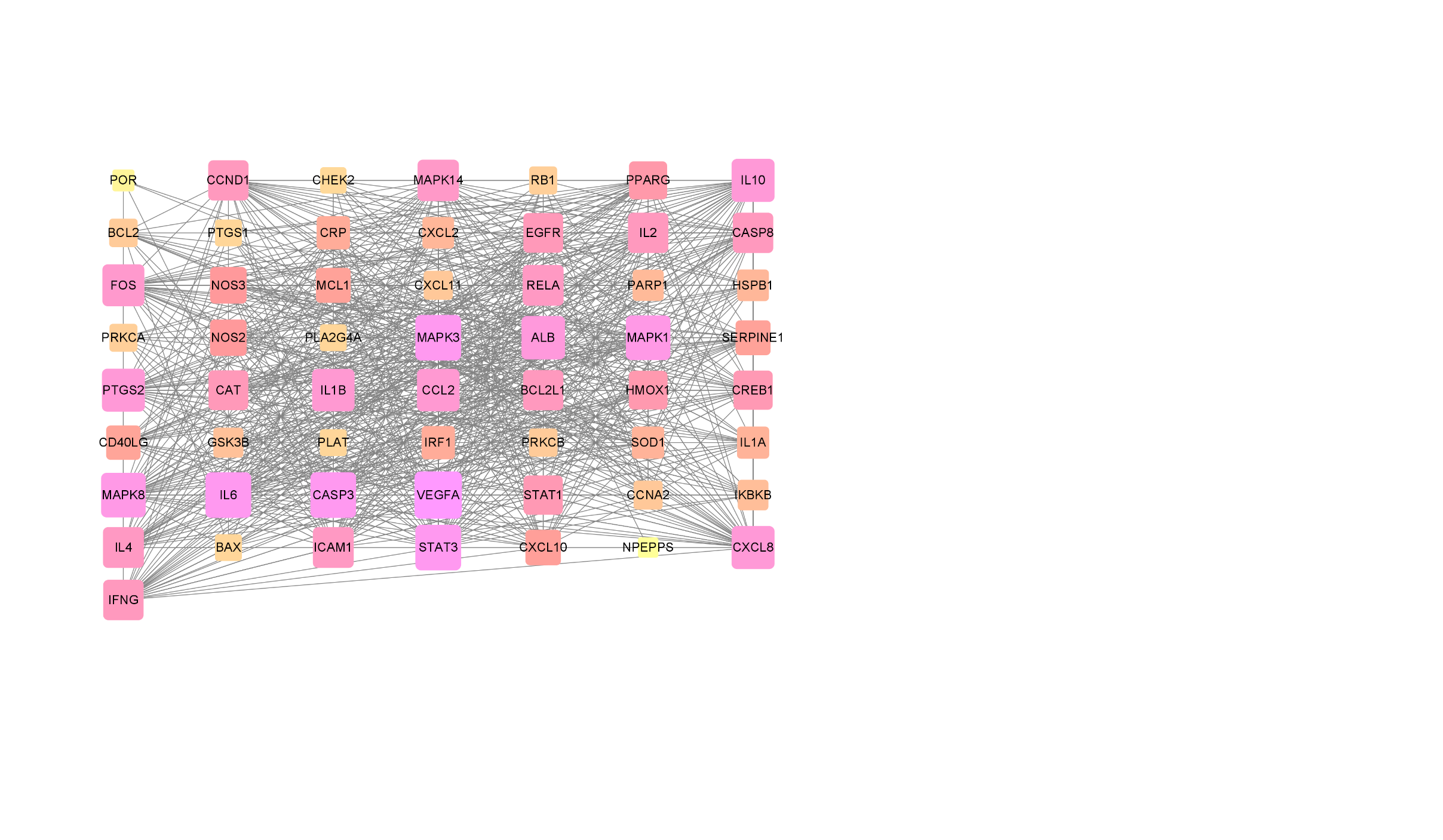


Fig. S4. The network of protein-protein interaction (PPI)


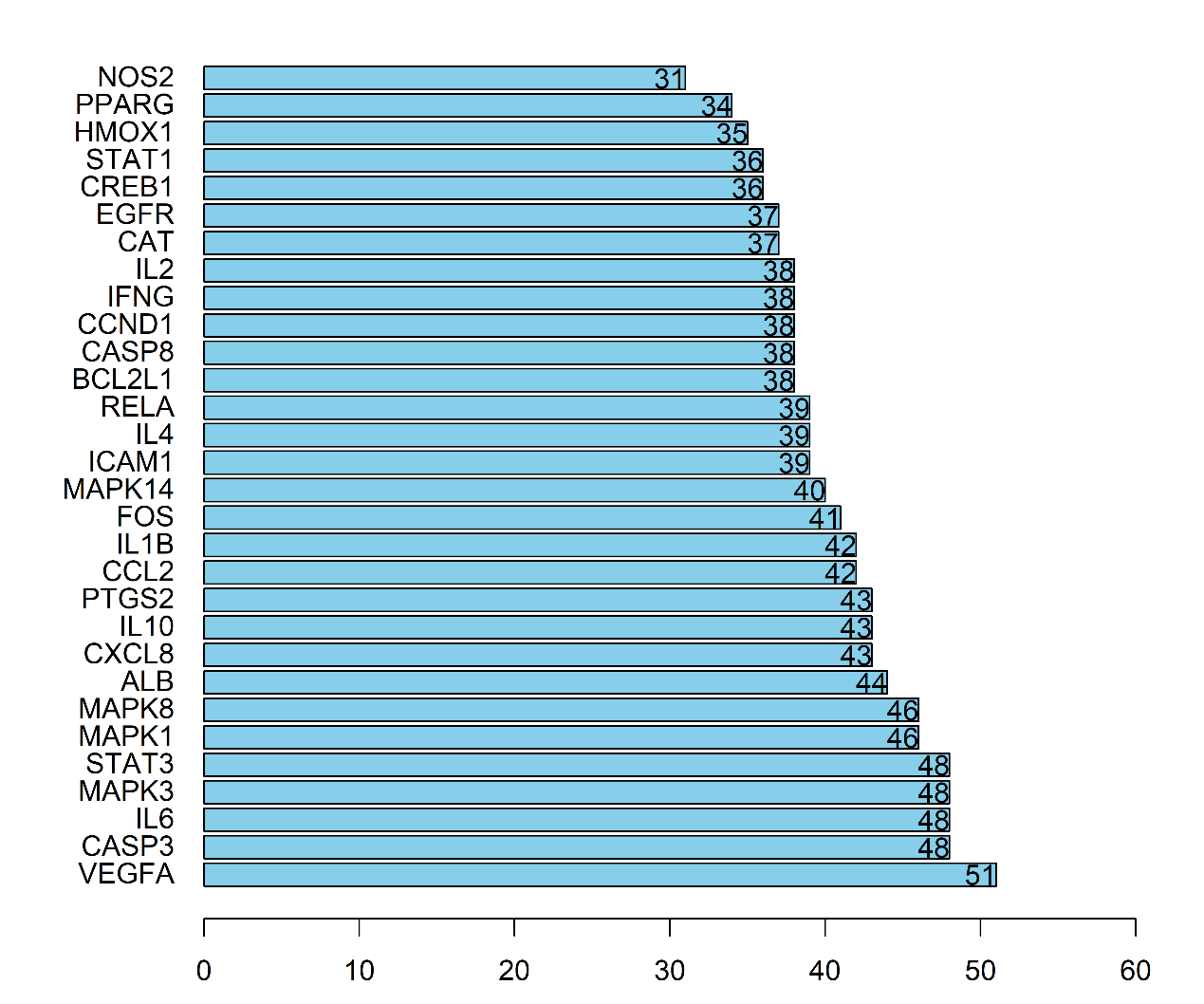


Fig. S5. Degree values of targets.

**Table S2 The informations of the tested compounds**

| No. | English name | Molecular formula | Molecular Weight | PubChem CID |
| --- | --- | --- | --- | --- |
| Positive Control | Ebselen | [C_13_H_9_NOSe](https://pubchem.ncbi.nlm.nih.gov/#query=C13H9NOSe) | 274.19 | 3194 |
| 1 | Hordenine | C10H15NO | 165.23 | 68313 |
| 2 | Herbacetin | C15H10O7 | 302.23 | 5280544 |
| 3 | Kaempferol | C15H10O6 | 286.24 | 5280863 |
| 4 | Quercetin | C15H10O7 | 302.23 | 5280343 |
| 5 | Naringenin | C15H12O5 | 272.25 | 932 |
| 6 | Hesperidin | C28H34O15 | 610.6 | 10621 |
| 7 | Tricin | C17H14O7 | 330.29 | 5281702 |
| 8 | Swertisin | C22H22O10 | 446.4 | 124034 |
| 9 | Vitexin | C21H20O10 | 432.4 | 5280441 |
| 10 | 4-Hydroxybenzoic acid | C7H6O3 | 138.12 | 135 |
| 11 | Syringic acid | C9H10O5 | 198.17 | 10742 |
| 12 | Ferulic acid | C10H10O4 | 194.18 | 445858 |
| 13 | Cis-p-Coumaric acid | C9H8O3 | 164.16 | 1549106 |
| 14 | Caffeic Acid | C9H8O4 | 180.16 | 689043 |
| 15 | Sinapic acid | C11H12O5 | 224.21 | 637775 |
| 16 | 2-Hydroxyphenylacetic acid | C8H8O3 | 152.15 | 11970 |
| 17 | Amygdalin | C20H27NO11 | 457.4 | 656516 |
| 18 | Atractyloside A | C21H36O10 | 448.5 | 71307451 |
| 19 | AtractylenolideⅠ | C15H18O2 | 230.3 | 5321018 |
| 20 | Aractylenolide Ⅱ | C15H20O2 | 232.32 | 14448070 |
| 21 | Atractylenolide Ⅲ | C15H20O3 | 248.32 | 155948 |
| 22 | Atractylon | C15H20O | 216.32 | 3080635 |
| 23 | Atractylodin | C13H10O | 182.22 | 5321047 |
| 24 | Pachypodol | C18H16O7 | 344.3 | 5281677 |
| 25 | Kumatakenin | C17H14O6 | 314.29 | 5318869 |
| 26 | Hyperoside | C21H20O12 | 464.4 | 5281643 |
| 27 | Epifriedelanol | C30H52O | 428.7 | 119242 |
| 28 | Friedelin | C30H50O | 426.7 | 91472 |
| 29 | Oleanolic acid | C30H48O3 | 456.7 | 10494 |
| 30 | Methyl oleanolate | C31H50O3 | 470.7 | 92900 |
| 31 | Artemisine | C15H22O5 | 282.33 | 68827 |
| 32 | Artemisitene | C15H20O5 | 280.32 | 11000442 |
| 33 | Dihydroartemisinic acid | C15H24O2 | 236.35 | 11020893 |
| 34 | Artemisinic acid | C15H22O2 | 234.33 | 10922465 |
| 35 | Chrysosplenol D | C18H16O8 | 360.3 | 5280699 |
| 36 | Chrysosplenetin | C19H18O8 | 374.3 | 5281608 |
| 37 | Blumeatin | C16H14O6 | 302.28 | 70696494 |
| 38 | Scopoletin | C10H8O4 | 192.17 | 5280460 |
| 39 | Scoparone | C11H10O4 | 206.19 | 8417 |
| 40 | Beta-Sitosterol | C_29_H_50_O | 414.7 | 222284 |
| 41 | Physcion | C_16_H_12_O_5_ | 284.26 | 10639 |
| 42 | 4-Hydroxybenzaldehyde | C_7_H_6_O_2_ | 122.12 | 126 |
| 43 | 5-Hydroxymethylfurfural | C6H6O3 | 126.11 | 237332 |
| 44 | Gentisuric acid | C9H9NO5 | 211.17 | 161488 |
| 45 | Coixol | C8H7NO3 | 165.15 | 10772 |
| 46 | Myrcene | C10H16 | 136.23 | 31253 |
| 47 | Linalool | C10H18O | 154.25 | 6549 |
| 48 | Naringin | C27H32O14 | 580.5 | 442428 |
| 49 | Rhoifolin | C27H30O14 | 578.5 | 5282150 |
| 50 | Isoimperatorin | C16H14O4 | 270.28 | 68081 |
| 51 | Meranzin | C15H16O4 | 260.28 | 1803558 |
| 52 | Isomeranzin | C15H16O4 | 260.28 | 473252 |
| 53 | Chrysophanol | C15H10O4 | 254.24 | 10208 |
| 54 | Physcion 1-glucoside | C22H22O10 | 446.4 | 168938 |
| 55 | Aloe-emodin | C15H10O5 | 270.24 | 10207 |
| 56 | Catechin | C15H14O6 | 290.27 | 9064 |
| 57 | Hesperetin | C16H14O6 | 302.28 | 72281 |
| 58 | Genistein | C15H10O5 | 270.24 | 5280961 |
| 59 | Gallic Acid | C7H6O5 | 170.12 | 370 |
| 60 | Hastatoside | C17H24O11 | 404.4 | 92043450 |
| 61 | Gentiopicroside | C16H20O9 | 356.32 | 88708 |
| 62 | Aucubin | C15H22O9 | 346.33 | 91458 |
| 63 | Kaempferide | C16H12O6 | 300.26 | 5281666 |
| 64 | Quercitrin | C21H20O11 | 448.4 | 5280459 |
| 65 | Oxalic Acid | C2H2O4 | 90.03 | 971 |
| 66 | 3-Epioleanolic acid | C30H48O3 | 456.7 | 11869658 |
| 67 | Verbenone | C10H14O | 150.22 | 92874 |
| 68 | Myristic acid | C14H28O2 | 228.37 | 11005 |
| 69 | Arachidic acid | C20H40O2 | 312.5 | 10467 |
| 70 | Linoleic Acid | C18H32O2 | 280.4 | 5280450 |
| 71 | Erucic acid | C22H42O2 | 338.6 | 5281116 |
| 72 | Isoquercitrin | C21H20O12 | 464.4 | 5280804 |
| 73 | Licochalcone D | C21H22O5 | 354.4 | 10473311 |
| 74 | Licochalcone C | C21H22O4 | 338.4 | 9840805 |
| 75 | Licochalcone B | C16H14O5 | 286.28 | 5318999 |
| 76 | Licochalcone E | C21H22O4 | 338.4 | 46209991 |
| 77 | Taxifolin | C15H12O7 | 304.25 | 439533 |
| 78 | Liquiritigenin | C15H12O4 | 256.25 | 114829 |
| 79 | Isoliquiritigenin | C15H12O4 | 256.25 | 638278 |
| 80 | Glabridin | C20H20O4 | 324.4 | 124052 |
| 81 | Glycyrrhizic acid | C42H62O16 | 822.9 | 14982 |
| 82 | Acteoside | C_29_H_36_O_15_ | 624.6 | 5281800 |
| 83 | Physcion | C_16_H_12_O_5_ | 284.26 | 10639 |


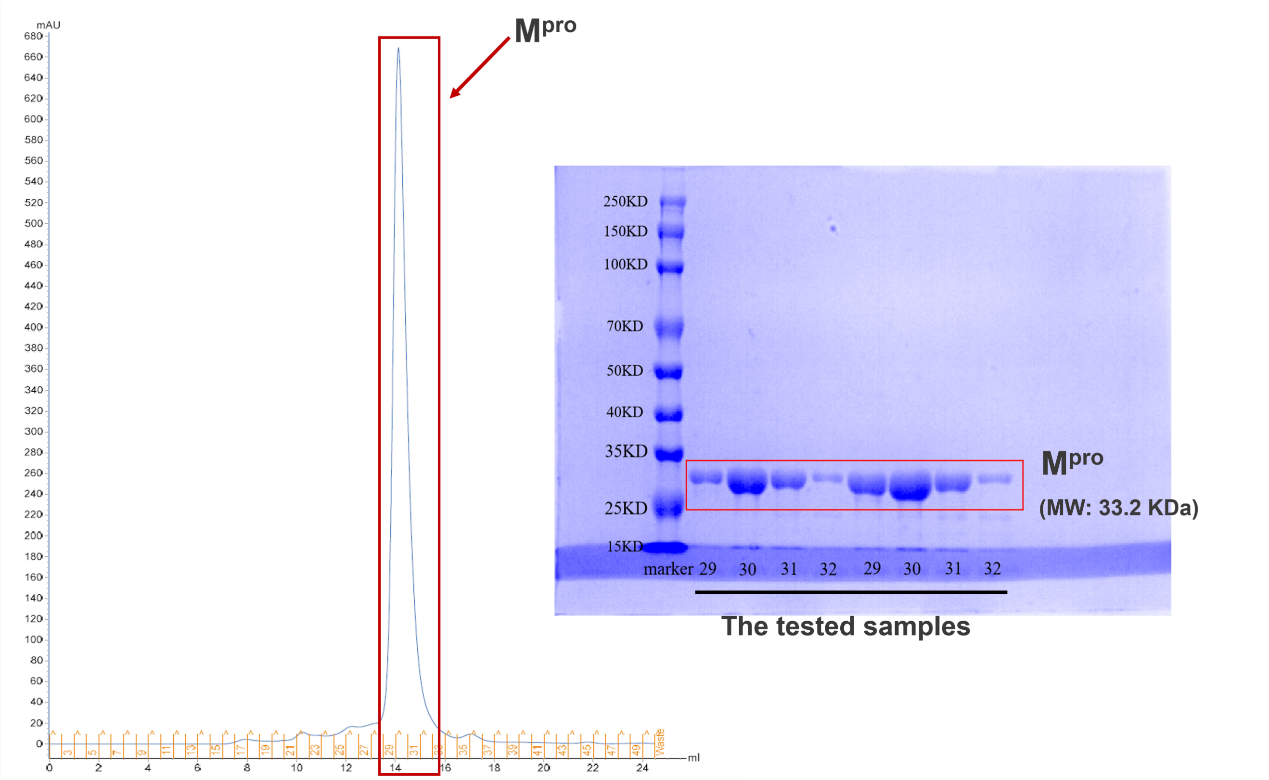


Fig. S6. The purified M^pro^ with the purity above 95%.


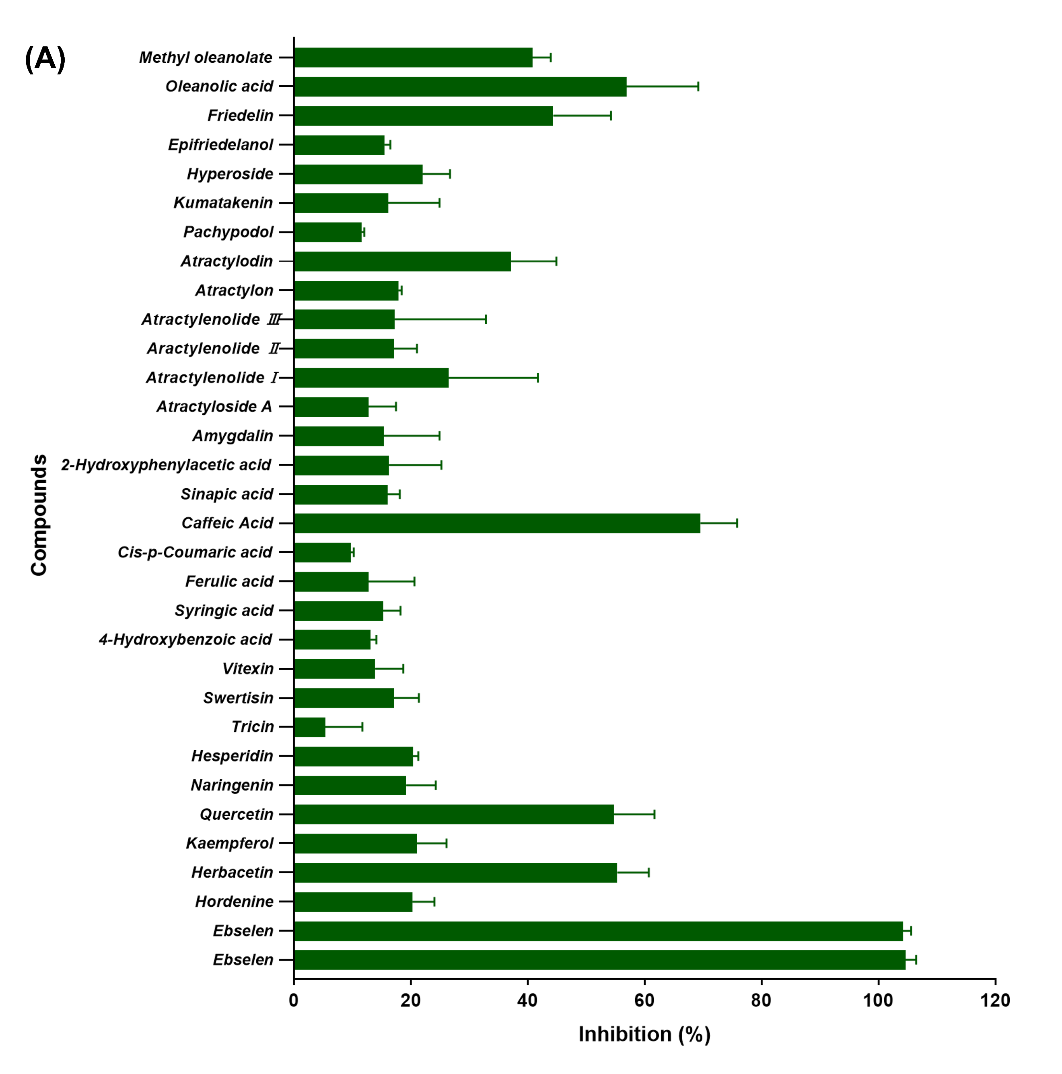


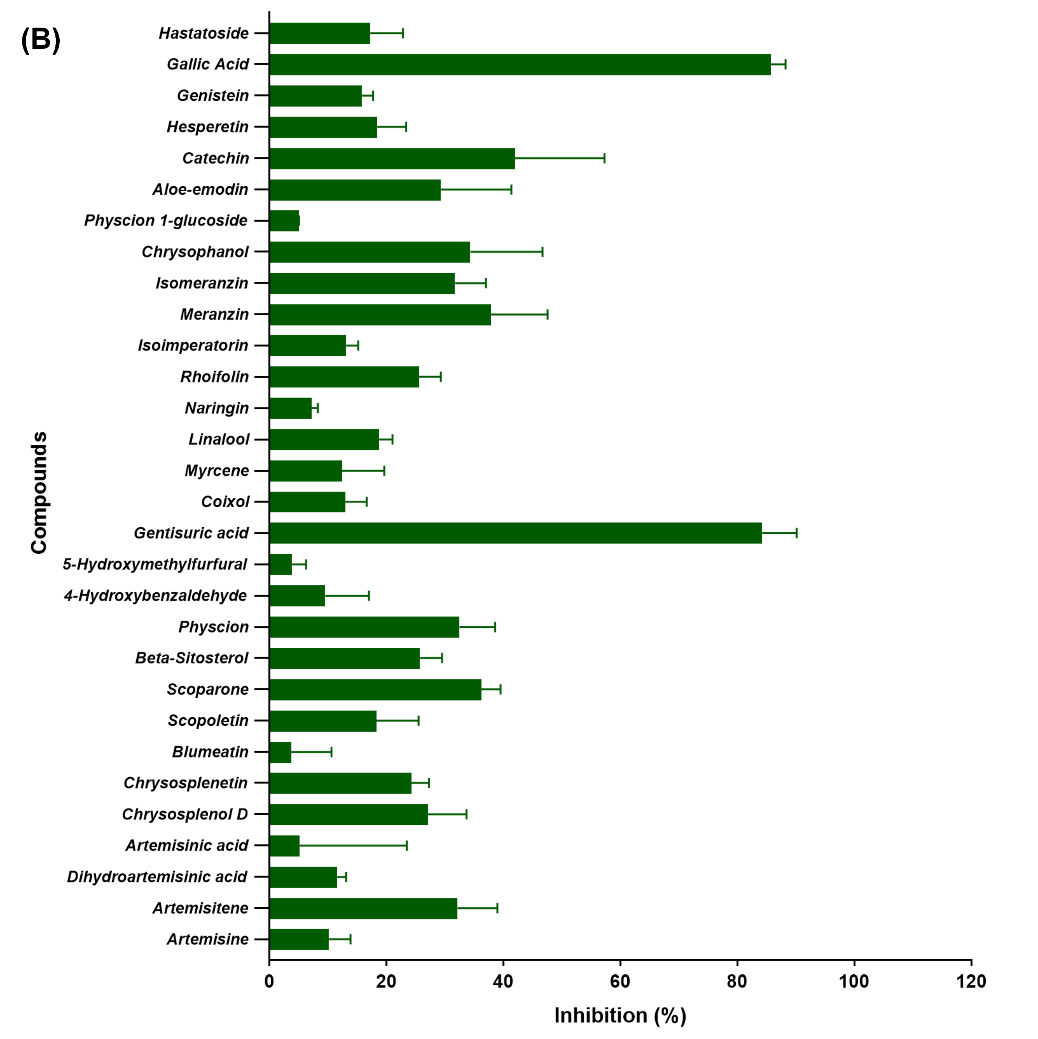


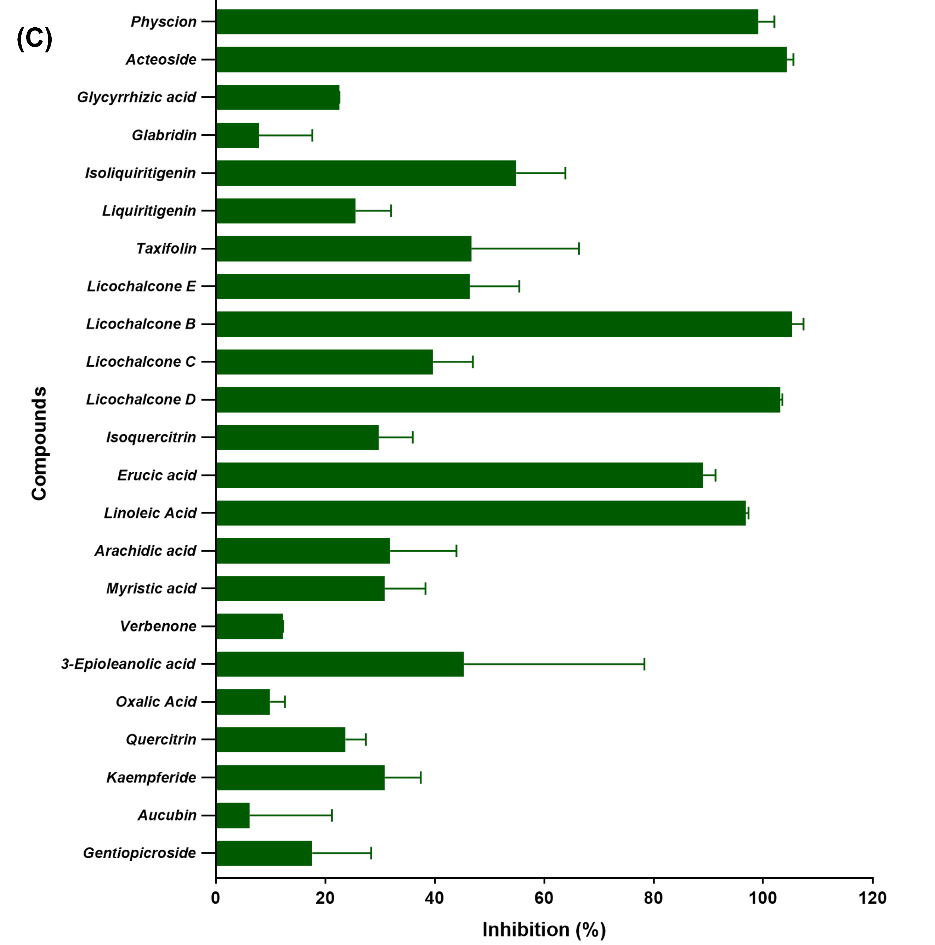


Fig. S7. The inhibition activity of the tested compounds on M^pro^ at 40 µM


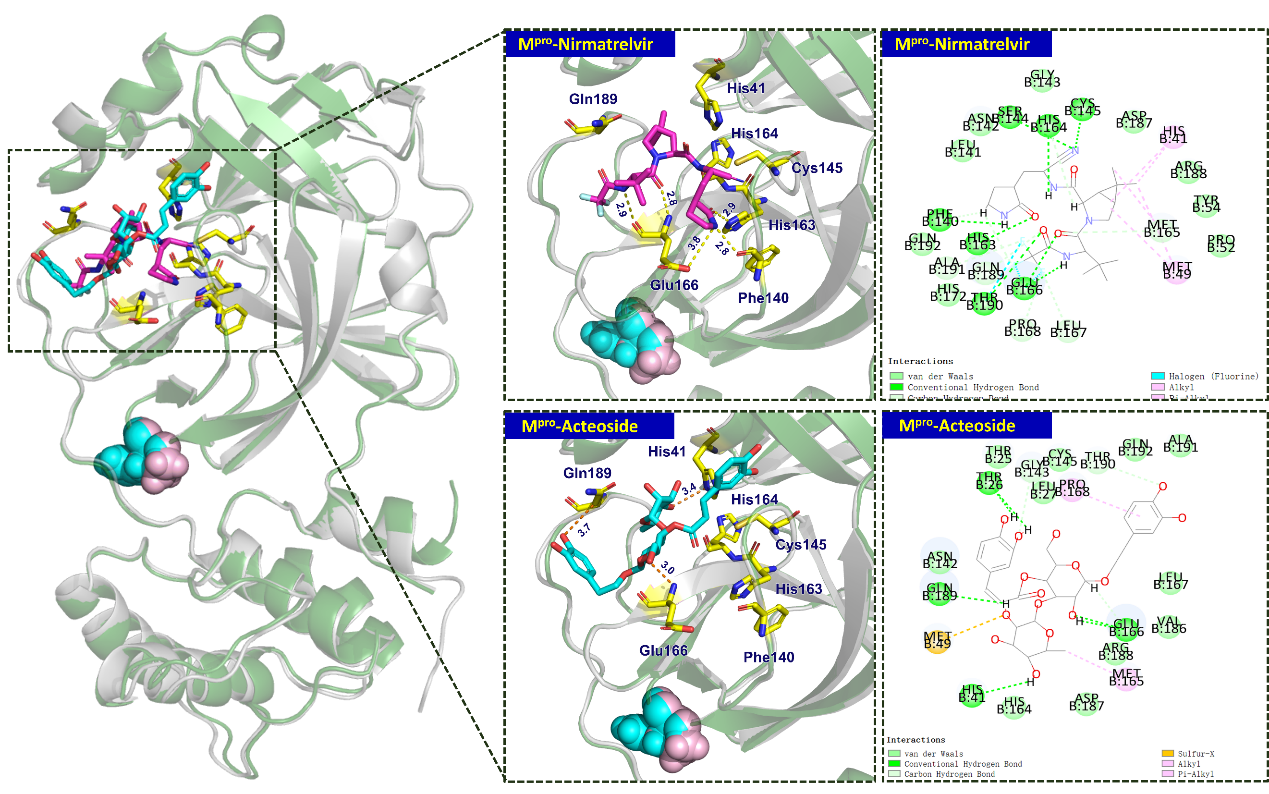


Fig. S8. The predicted structure of the acteoside-M^pro^ P132H and Nirmatrelvir-M^pro^ P132H complexes. WT M^pro^ His132 (*gray and blue*) and M^pro^ P132H (*green and pink*) are superposed.


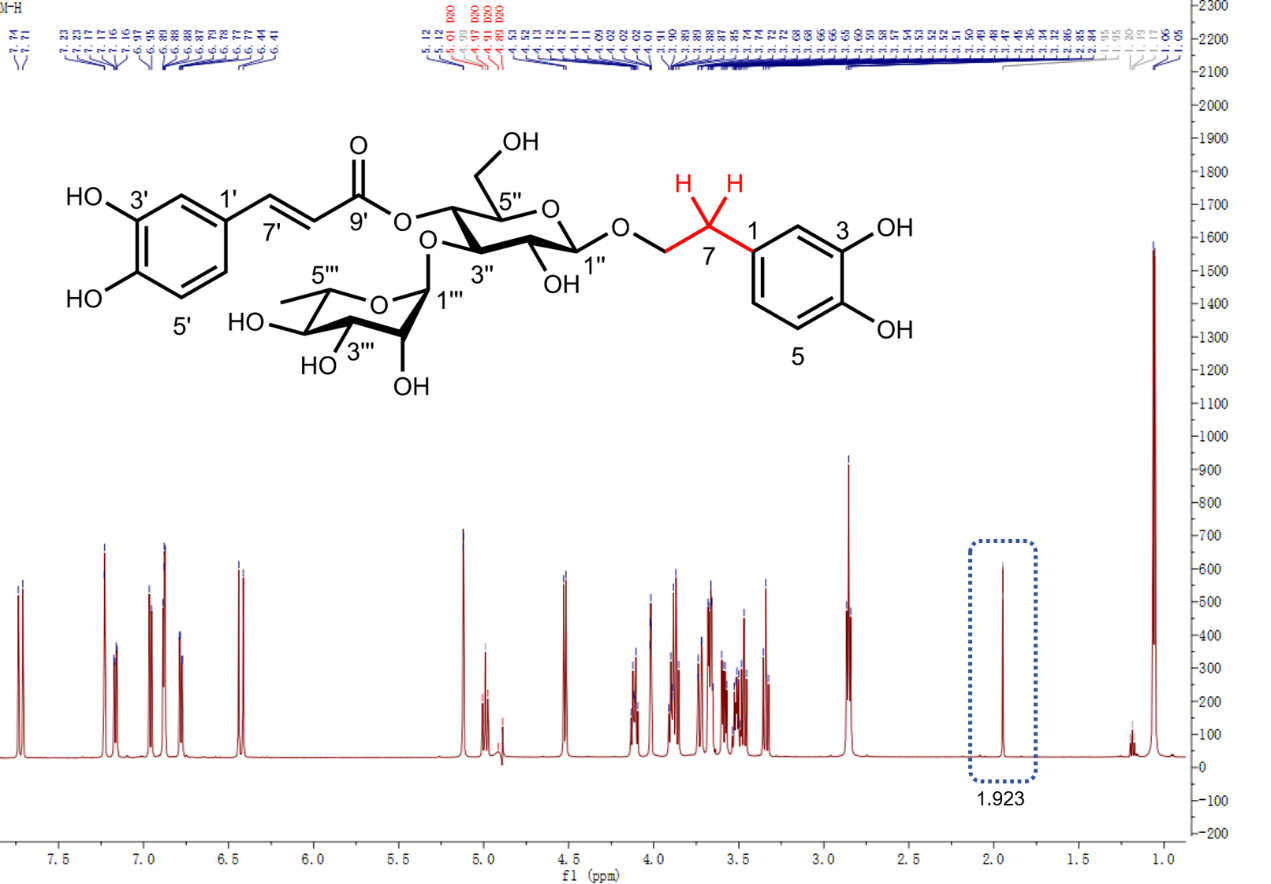


Fig. S9. ^1^H NMR spectrum of acteoside
